# Supplementary material for: Cysteine and Folate Metabolism Are Targetable Vulnerabilities of Metastatic Colorectal Cancer
Source: Cancers (Basel). 2021 Jan 23;13(3):425. doi: 10.3390/cancers13030425 (PMC7866204; doi:10.3390/cancers13030425)

Supplementary Files

Supplementary Tables

	v (nmol/(10^{6}cell volume·h))					
**Cell line**	**SW480**		**SW620**		**LiM2**	
**Amino acid**	**Mean**	**SD**	**Mean**	**SD**	**Mean**	**SD**
**Alanine**	17,39	2,9	24,64	0,59	24,86	0,88
**Glycine**	7,31	2,56	6,54	1,99	6,32	2,76
**Serine**	-6,04	3,02	-15,31	0,55	-13,02	0,70
**Proline**	2,72	0,18	5,00	0,36	5,18	0,37
**Arginine**	3,42	5,63	-4,36	0,43	-2,57	1,35
**Asparagine**	2,79	0,30	0,49	0,04	0,42	0,02
**Aspartate**	1,24	0,24	0,48	0,03	0,55	0,04
**Glutamine**	-59,89	23,48	-86,01	5,35	-77,2	9,16
**Glutamate**	9,13	1,10	8,76	0,97	5,64	0,78
**Methionine**	-0,92	0,88	0,53	0,44	-1,32	1,02
**Histidine**	-1,51	1,17	-0,03	0,13	-1,09	0,74
**Isoleucine**	-3,04	4,89	-0,08	2,05	-4,93	3,06
**Leucine**	-3,30	2,09	-2,63	1,62	-6,43	2,26
**Threonine**	-3,31	5,37	1,67	2,23	-1,14	3,41
**Tyrosine**	4,03	1,11	1,49	0,77	-0,47	1,67
**Valine**	5,80	2,91	2,99	3,08	-4,08	1,66
**Phenylalanine**	1,30	1,05	0,82	0,25	-2,09	1,05
**Lysine**	3,51	3,96	-0,07	0,47	-2,69	1,69
**Tryptophan**	1,52	1,08	-0,08	0,06	-0,93	0,29
**Taurine**	-0,4606	0,0234	-0,2269	0,0053	-0,1621	0,0179

**Spermidine**	0,0012	0,0006	0,0004	0,0002	0,0148	0,0019
**Putrescine**	0,0019	0,0003	0,0086	0,0011	0,0075	0,0001

Table S1. Amino acids uptake and production upon metastatic progression. Amino acids and other biogenic amines (in bold) uptake and production rates obtained by measuring the cell culture medium with an Absolute IDQ p180 kit (Biocrates Life Sciences AG) before and after 24 hours of incubation with DMEM 12.5 mM glucose and 4 mM glutamine, 5% FBS and 1% S/P, normalised by cell volume for each cell line (in bold). Mean and standard deviation (SD) are represented (in bold).

Gene symbols		The predicted fraction of growth compared to wild type		
		SW480	SW620	LiM2
Single Targets	***MTHFD1***	100.0%	0.0%	0.0%
	GSR	99.0%	0.0%	0.0%
	SCD	25.4%	0.0%	0.0%
	DLD	5.5%	0.0%	3.5%
	PKM	2.3%	0.0%	0.0%
	PRODH	0.0%	0.0%	0.0%
	GAPDH	0.0%	0.0%	0.0%
	GPT	0.0%	0.0%	0.0%
	RRM1	0.0%	0.0%	0.0%
	GUK1	0.0%	0.0%	0.0%
Target pairs	***SLC7A9, SLC3A2***	86%	0%	0%
	SLC3A1, SLC3A2	86%	0%	0%
	SLC7A9, SLC7A11	85%	0%	0%
	SLC7A11, SLC3A1	85%	0%	0%
	ETFA, GPI	49%	10%	9%
	ETFB, GPI	49%	10%	9%
	AGXT, GPI	49%	9%	8%
	GPI, GRHPR	49%	9%	8%
	CTH, GPI	42%	4%	7%
	DLST, GPI	40%	5%	7%
	GPI, OGDH	40%	5%	7%

	GPI, SUCLG1	35%	1%	1%
	G6PD, GPI	24%	2%	2%
	PGLS, GPI	24%	2%	2%
	FH, GPI	19%	2%	7%
	PPA2, GPI	18%	0%	0%
	GPI, PGD	16%	0%	0%
	GPI, SDHD	14%	0%	0%
	GPI, SDHC	14%	0%	0%
	GPI, SDHA	14%	0%	0%
	GPI, SDHB	14%	0%	0%
	GPI, UQCRH	12%	0%	0%
	UQCRQ, GPI	12%	0%	0%
	GPI, UQCRFS1	12%	0%	0%
	UQCR11, GPI	12%	0%	0%
	GPI, UQCR10	12%	0%	0%
	GPI, MT-CYB	12%	0%	0%
	CYC1, GPI	12%	0%	0%
	GPI, UQCRC1	12%	0%	0%
	GPI, UQCRB	12%	0%	0%
	GPI, UQCRC2	12%	0%	0%
	FH, PDHB	6%	8%	9%
	DLAT, FH	6%	8%	9%
	FH, ATP5J	0%	0%	0%

	MT-CO1, SDHA	0%	4%	0%
	COX4I1, SDHA	0%	4%	0%
	CYC1, ACOX1	0%	0%	0%
	SDHD, COX5A	0%	4%	0%
	NSF, SDHD	0%	0%	0%
	COX4I1, SDHD	0%	4%	0%
	COX7B2, SDHA	0%	4%	0%
	COX4I1, SDHC	0%	4%	0%
	COX7B, SDHC	0%	4%	0%
	ATP5B, SDHA	0%	0%	0%
	SDHC, COX4I2	0%	4%	0%
	COX6B2, SDHD	0%	4%	0%
	COX8A, ACOX1	0%	0%	0%
	ATP5C1, SDHA	0%	0%	0%
	GPI, SORD	0%	0%	0%
	MT-CYB, ACOX1	0%	0%	0%
	COX7B, GPI	0%	0%	0%
	MT-CO2, ACOX1	0%	0%	0%
	COX5B, SDHD	0%	4%	0%
	ATP5H, SDHC	0%	0%	0%
	COX7C, ACOX1	0%	0%	0%
	UQCRQ, ACOX1	0%	0%	0%
	ACOX1, UQCRH	0%	0%	0%

	GPI, COX8C	0%	0%	0%
	ATP5H, GPI	0%	0%	0%
	GPI, ATP5C1	0%	0%	0%
	ATP5L, SDHA	0%	0%	0%
	GPI, ATP5F1	0%	0%	0%
	COX7B, ACOX1	0%	0%	0%
	ATP5D, SDHB	0%	0%	0%
	GPI, ATP5D	0%	0%	0%
	SDHC, COX5A	0%	4%	0%
	ATP5E, SDHC	0%	0%	0%
	COX7A2, GPI	0%	0%	0%
	COX7B2, ACOX1	0%	0%	0%
	SDHA, ATP5J2	0%	0%	0%
	COX7A2, SDHB	0%	4%	0%
	COX7C, SDHC	0%	4%	0%
	COX6B1, SDHD	0%	4%	0%
	COX6B1, SDHB	0%	4%	0%
	COX6B2, SDHC	0%	4%	0%
	GPI, COX5A	0%	0%	0%
	COX7A2, ACOX1	0%	0%	0%
	GPI, ATP5A1	0%	0%	0%
	MT-CO2, SDHD	0%	4%	0%
	ATP5I, SDHC	0%	0%	0%

	GPI, MT-CO2	0%	0%	0%
	COX7A1, SDHD	0%	4%	0%
	FH, ATP5D	0%	0%	0%
	FH, ATP5C1	0%	0%	0%
	COX5B, SDHC	0%	4%	0%
	GPI, ATP5J	0%	0%	0%
	ACOX1, UQCRB	0%	0%	0%
	COX7B2, SDHD	0%	4%	0%
	COX6C, SDHB	0%	4%	0%
	SDHD, COX7A2L	0%	4%	0%
	SDHC, ATP5J2	0%	0%	0%
	ACOX1, UQCRC1	0%	0%	0%
	ATP5L, SDHB	0%	0%	0%
	ACOX1, UQCRC2	0%	0%	0%
	ATP5J, SDHC	0%	0%	0%
	ATP5E, SDHA	0%	0%	0%
	COX7A2, SDHC	0%	4%	0%
	COX6A1, SDHC	0%	4%	0%
	COX7C, SDHD	0%	4%	0%
	ATP5D, SDHD	0%	0%	0%
	COX6A1, GPI	0%	0%	0%
	COX7B, SDHA	0%	4%	0%
	COX6A2, GPI	0%	0%	0%

	ATP5A1, SDHD	0%	0%	0%
	GPI, NSF	0%	0%	0%
	ACOX1, COX7A2L	0%	0%	0%
	UQCR11, ACOX1	0%	0%	0%
	COX6A1, ACOX1	0%	0%	0%
	SDHB, COX4I2	0%	4%	0%
	COX8C, SDHC	0%	4%	0%
	ATP5H, SDHA	0%	0%	0%
	COX7A1, SDHC	0%	4%	0%
	ATP5I, SDHA	0%	0%	0%
	MT-CO2, SDHB	0%	4%	0%
	SDHC, COX7A2L	0%	4%	0%
	FH, ATP5I	0%	0%	0%
	COX7A1, GPI	0%	0%	0%
	ATP5D, SDHA	0%	0%	0%
	FH, ATP5F1	0%	0%	0%
	COX7B2, SDHB	0%	4%	0%
	MT-CO3, SDHC	0%	4%	0%
	ATP5F1, SDHC	0%	0%	0%
	COX6A1, SDHA	0%	4%	0%
	COX6C, GPI	0%	0%	0%
	COX6C, ACOX1	0%	0%	0%
	NSF, SDHC	0%	0%	0%

	ATP5J, SDHA	0%	0%	0%
	COX8A, GPI	0%	0%	0%
	COX6A2, SDHC	0%	4%	0%
	ATP5F1, SDHA	0%	0%	0%
	COX6B1, SDHA	0%	4%	0%
	MT-CO1, SDHD	0%	4%	0%
	ATP5A1, SDHB	0%	0%	0%
	COX5B, GPI	0%	0%	0%
	MT-CO1, SDHB	0%	4%	0%
	ATP5C1, SDHD	0%	0%	0%
	FH, ATP5E	0%	0%	0%
	MT-CO2, SDHA	0%	4%	0%
	SDHA, COX5A	0%	4%	0%
	COX7A2, SDHD	0%	4%	0%
	COX8C, ACOX1	0%	0%	0%
	MT-CO3, SDHA	0%	4%	0%
	GPI, ATP5B	0%	0%	0%
	GPI, COX7A2L	0%	0%	0%
	NSF, SDHA	0%	0%	0%
	ATP5L, FH	0%	0%	0%
	MT-CO3, ACOX1	0%	0%	0%
	COX4I1, ACOX1	0%	0%	0%
	ATP5C1, SDHC	0%	0%	0%

	SDHA, COX7A2L	0%	4%	0%
	COX7B, SDHD	0%	4%	0%
	COX8A, SDHC	0%	4%	0%
	SDHD, ATP5J2	0%	0%	0%
	COX7A1, ACOX1	0%	0%	0%
	COX6B2, SDHA	0%	4%	0%
	COX7C, GPI	0%	0%	0%
	COX8C, SDHA	0%	4%	0%
	FH, ATP5B	0%	0%	0%
	ATP5H, SDHD	0%	0%	0%
	GPI, COX4I2	0%	0%	0%
	ATP5L, SDHD	0%	0%	0%
	ATP5H, SDHB	0%	0%	0%
	COX6C, SDHD	0%	4%	0%
	ACOX1, COX4I2	0%	0%	0%
	GPI, ATP5E	0%	0%	0%
	COX6A1, SDHD	0%	4%	0%
	COX6B2, ACOX1	0%	0%	0%
	ACOX1, COX5A	0%	0%	0%
	ATP5D, SDHC	0%	0%	0%
	GPI, ATP5J2	0%	0%	0%
	COX6B1, ACOX1	0%	0%	0%
	SDHB, COX7A2L	0%	4%	0%

	ATP5E, SDHB	0%	0%	0%
	COX7B, SDHB	0%	4%	0%
	COX7C, SDHB	0%	4%	0%
	COX6B1, SDHC	0%	4%	0%
	COX6A2, SDHA	0%	4%	0%
	ATP5B, SDHC	0%	0%	0%
	FH, ATP5J2	0%	0%	0%
	COX7A1, SDHA	0%	4%	0%
	ATP5I, SDHB	0%	0%	0%
	MT-CO2, SDHC	0%	4%	0%
	GPI, MT-CO1	0%	0%	0%
	FH, ATP5A1	0%	0%	0%
	COX5B, SDHB	0%	4%	0%
	GPI, ATP5I	0%	0%	0%
	ACOX1, UQCRFS1	0%	0%	0%
	COX7A2, SDHA	0%	4%	0%
	COX4I1, GPI	0%	0%	0%
	COX6C, SDHC	0%	4%	0%
	ATP5H, FH	0%	0%	0%
	SDHD, COX4I2	0%	4%	0%
	MT-CO3, SDHD	0%	4%	0%
	ATP5F1, SDHD	0%	0%	0%
	ATP5L, SDHC	0%	0%	0%

	SDHB, COX5A	0%	4%	0%
	ATP5J, SDHB	0%	0%	0%
	FH, NSF	0%	0%	0%
	COX6A1, SDHB	0%	4%	0%
	ATP5B, SDHD	0%	0%	0%
	UQCR10, ACOX1	0%	0%	0%
	COX5B, SDHA	0%	4%	0%
	ATP5A1, SDHA	0%	0%	0%
	COX8A, SDHA	0%	4%	0%
	COX5B, ACOX1	0%	0%	0%
	COX8C, SDHD	0%	4%	0%
	COX8C, SDHB	0%	4%	0%
	COX6A2, ACOX1	0%	0%	0%
	SDHB, ATP5J2	0%	0%	0%
	COX7A1, SDHB	0%	4%	0%
	COX6B2, SDHB	0%	4%	0%
	ATP5I, SDHD	0%	0%	0%
	ATP5C1, SDHB	0%	0%	0%
	COX6C, SDHA	0%	4%	0%
	COX7B2, SDHC	0%	4%	0%
	COX7B2, GPI	0%	0%	0%
	MT-CO3, SDHB	0%	4%	0%
	NSF, SDHB	0%	0%	0%

	COX4I1, SDHB	0%	4%	0%
	ATP5F1, SDHB	0%	0%	0%
	ATP5L, GPI	0%	0%	0%
	ATP5J, SDHD	0%	0%	0%
	COX7C, SDHA	0%	4%	0%
	ATP5E, SDHD	0%	0%	0%
	SDHA, COX4I2	0%	4%	0%
	COX6B1, GPI	0%	0%	0%
	COX6A2, SDHD	0%	4%	0%
	COX6A2, SDHB	0%	4%	0%
	ATP5B, SDHB	0%	0%	0%
	MT-CO1, SDHC	0%	4%	0%
	COX8A, SDHB	0%	4%	0%
	COX8A, SDHD	0%	4%	0%
	ATP5A1, SDHC	0%	0%	0%
	COX6B2, GPI	0%	0%	0%
	MT-CO1, ACOX1	0%	0%	0%
	GPI, MT-CO3	0%	0%	0%

Table S2. Putative metabolic targets. List of the individual or targets pairs (in bold) identified in the model that can impair SW620 and LiM2 proliferation (cell lines in bold).

Inhibitor	IC_{50} values			
	NCM460	SW480	SW620	LiM2
**Sulfasalazine (μM)**	518 ± 43[a]	534 ± 58[a]	429 ± 66[b]	415 ± 56[b]
**Erastin (μM)**	1.35 ± 0.32[a]	1.00 ± 0.05[b]	0.86 ± 0.27[c]	0.52 ± 0.13[d]
**2-AAPA (μM)**	175 ± 76[a]	61 ± 38[b]	28 ± 10[c]	20 ± 2[c]
**LY345899 (μM)**	191.1 ± 9.9[a]	178.3 ± 45.3[b]	57.5 ± 9.2[c]	58.1 ± 12.7[c]
**SHIN2 (μM)**	55.9 ± 5.7[a]	19.8 ± 3.5[b]	19.7 ± 3.3[b]	20.8 ± 4.2[b]
**Methotrexate (nM)**	72.8 ± 5.2[a]	14.9 ± 2.5[b]	10.9 ± 3.4[c]	9.7 ± 2.3[c]

Table S3. IC_{50} values for the tested inhibitors. List of tested inhibitors (in bold) and the IC_{50} values for NCM460, SW480, SW620 and LiM2 cell lines (in bold). The IC_{50} curves were performed assessing cell proliferation by staining DNA with HO33342 under various concentrations of the inhibitors. Sulfasalazine and erastin are specific inhibitor of system xCT, 2-AAPA is an specific inhibitor of GSR (glutathione reductase), LY345899 is an specific inhibitor of MTHFD1/2 (methylenetetrahydrofolate dehydrogenase, cyclohydrolase and formyltetrahydrofolate synthetase 1/2), SHIN2 is an specific inhibitor of SHMT1/2 (serine hydroxymethyltransferase 1/2), methotrexate is an inhibitor of DHFR (dihydrofolate reductase). [a,b,c,d] A one-way ANOVA and Scheffe's test for multiple comparisons was performed for the factor "cell line".

Cell line	CI values [Erastin] + [2-AAPA]			
**Dose (µM)**	**0.125 + 5**	**0.25 + 10**	**0.38 + 15**	**0.88 + 35**
**NCM460**	1.43	1.17	1.46	1.56
**SW480**	0.91	0.81	0.36	1.24
**SW620**	1.21	1.36	1.24	0.06
**LiM2**	1.28	2.56	0.56	0.52
**Cell line**	**CI values [Erastin] + [LY345899]**			
**Dose (µM)**	**0.25 + 10**	**0.5 + 20**	**1.25 + 50**	**1.88 + 75**
**NCM460**	1.21	1.42	1.07	1.14
**SW480**	1.18	1.93	1.46	1.27
**SW620**	1.35	1.19	0.43	0.31
**LiM2**	1.41	0.63	0.45	0.22

Table S4. Combination index values for the tested combinations. List of the combination index (CI) values calculated with CompuSyn, Inc, software (Chou-Talalay's CI method) of the following combinations (in bold): Erastin + 2AAPA at 120h, adding 2AAPA after 72h of Erastin treatment alone, erastin + LY345899 at 72h,. Combinations were tested in cell lines (in bold) NCM460, SW480, SW620 and LiM2. Erastin is an specific inhibitor of system xCT, 2-AAPA is an specific inhibitor of GSR (glutathione reductase), LY345899 is an specific inhibitor of MTHFD1/2 (methylenetetrahydrofolate dehydrogenase, cyclohydrolase and formyltetrahydrofolate synthetase 1/2). CI<1, CI=1 and CI>1 indicates synergy, additive effect and antagonism, respectively.

Isobaric species	Most probable acyl chains	SW480 (nmol/mg prot)	SW620 (nmol/mg prot)	LiM2 (nmol/mg prot)
**PC aa C34:1**	C16:0 + C18:1	207.886	345.588	293.647
**PC aa C34:2**	C16:1 + C18:1	56.178	90.967	79.069
**PC aa C36:1**	C:18 + C18:1	30.288	44.433	40.421
**PC aa C36:2**	C18:1 + C18:1	97.877	168.484	145.409

Table S5. Levels of the most abundant phosphatidylcholines (PC AA) and the most probable acyl chain for each of the most abundant phosphatidylcholines. PC AA were obtained by measuring the intracellular content by an Absolute IDQ p180 kit (Biocrates Life Sciences AG) after incubation with DMEM 12.5 mM Glc and 4 mM Gln, 5% FBS and 1% S/P, normalized by protein content for each cell line (in bold).

Acyl chain	SW480	SW620	LiM2
**C16:0**	30.5%	30.3%	29.9%
**C16:1**	14.3%	13.8%	13.6%
**C18:0**	3.0%	2.9%	3.1%
**C18:1**	52.2%	53.0%	53.4%

Table S6. Predicted relative abundance of each acyl chain in each cell line. These abundances were inferred from the relative abundance of phosphatidylcholines (Table S5) and used to define the acyl-CoA requirements for biomass synthesis in each cell line.

Supplementary Figures

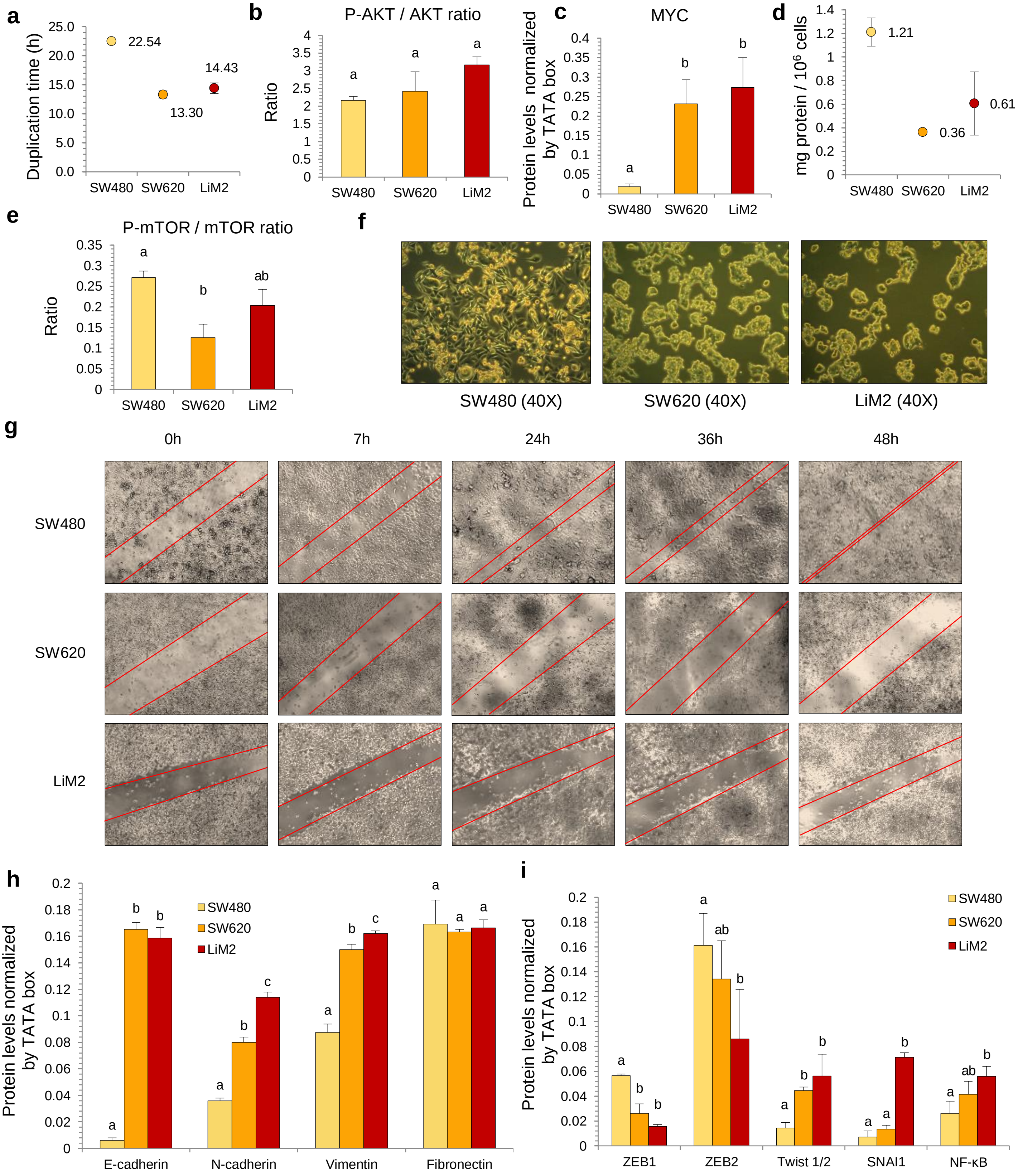
a
Duplication time (h)
22.54
13.30
14.43
SW480 SW620 LiM2
b
P-AKT / AKT ratio
Ratio
SW480 SW620 LiM2
c
MYC
Protein levels normalized by TATA box
SW480 SW620 LiM2
d
mg protein / 10^6 cells
1.21
0.36
0.61
SW480 SW620 LiM2
e
P-mTOR / mTOR ratio
Ratio
SW480 SW620 LiM2
f
SW480 (40X)
SW620 (40X)
LiM2 (40X)
g
0h
7h
24h
36h
48h
SW480
SW620
LiM2
h
Protein levels normalized by TATA box
SW480
SW620
LiM2
E-cadherin
N-cadherin
Vimentin
Fibronectin
i
Protein levels normalized by TATA box
SW480
SW620
LiM2
ZEB1
ZEB2
Twist 1/2
SNAI1
NF-κB

Figure S1

Figure S1. Characterisation of the metastatic phenotype. Related to Figure 1. (**a**) Average doubling time from multiple growth curves. (**b**) P-AKT / AKT ratio from the experiment in Figure 1b. (**c**) MYC protein level quantification normalised by TATA box protein levels. (**d**) Protein content measured by BCA assay related to cell number. (**e**) P-mTOR / mTOR ratio from the experiment in Fig. 1B normalised by TATA box protein levels. (**f**) Images of the cells at maximum confluence with contrast-phase microscope (40X). (**g**) Wound healing assay images with contrast-phase microscope (40X). (**h**) Protein level quantification from the experiment in Fig. 1G normalised by TATA box protein levels. (**i**) Protein level quantification from the experiment in Fig. 1H normalised by TATA box protein levels. [a,b,c] A one-way ANOVA was performed for the factor "cell line", and Scheffe's test was used for multiple comparisons. Groups sharing the same letter do not show a significant difference with α=0.05.

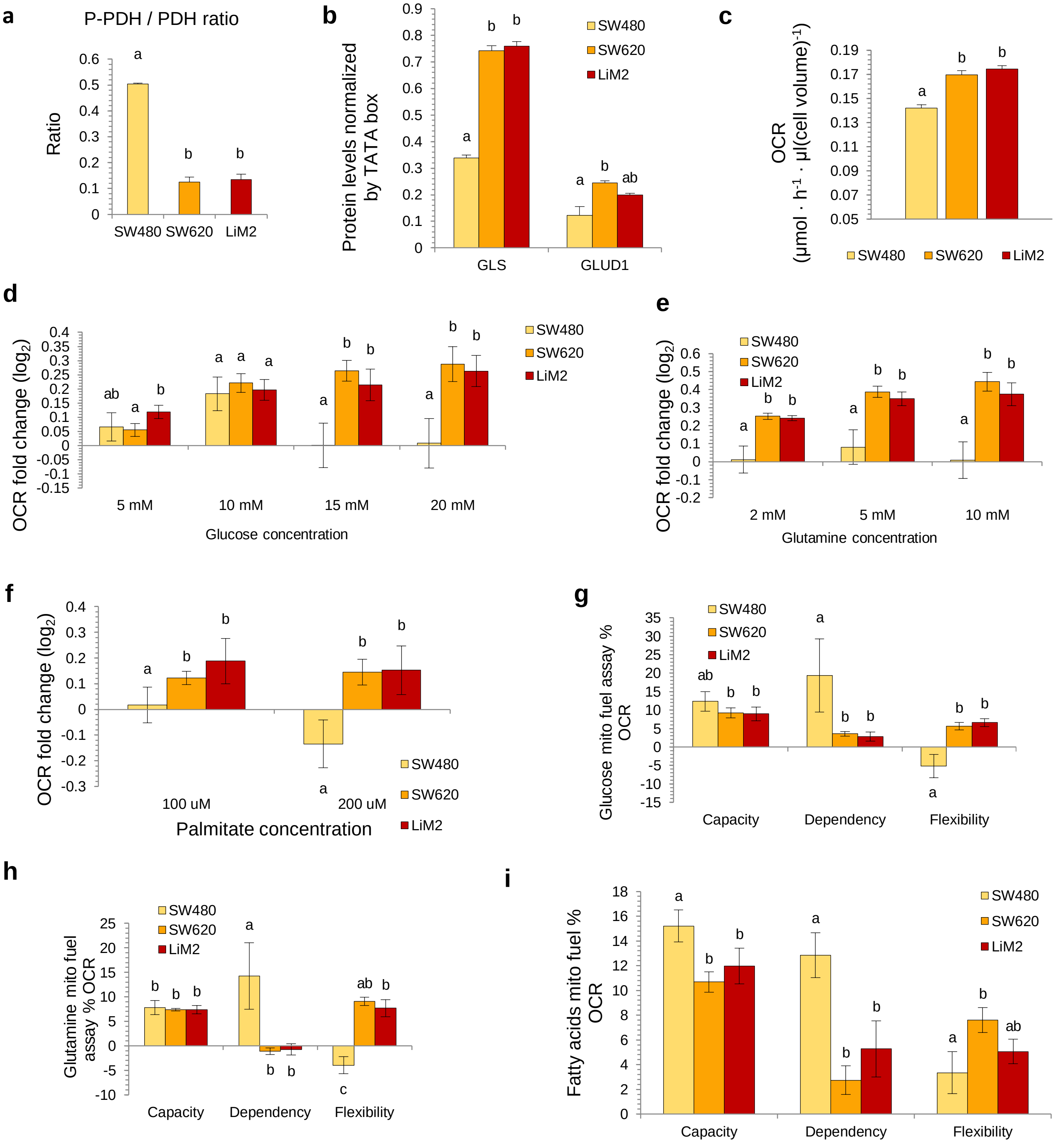
a
P-PDH / PDH ratio
Ratio
0.6
0.5
0.4
0.3
0.2
0.1
0
a
b
b
SW480 SW620 LiM2
b
Protein levels normalized by TATA box
0.9
0.8
0.7
0.6
0.5
0.4
0.3
0.2
0.1
0
a
b
b
a
b
ab
GLS
GLUD1
SW480
SW620
LiM2
c
OCR (µmol · h-1 · µl(cell volume)-1)
0.19
0.17
0.15
0.13
0.11
0.09
0.07
0.05
a
b
b
SW480
SW620
LiM2
d
OCR fold change (log2)
0.4
0.35
0.3
0.25
0.2
0.15
0.1
0.05
0
-0.05
-0.1
-0.15
ab
a
b
a
a
a
a
b
b
a
b
b
5 mM
10 mM
15 mM
20 mM
Glucose concentration
SW480
SW620
LiM2
e
OCR fold change (log2)
0.6
0.5
0.4
0.3
0.2
0.1
0
-0.1
-0.2
a
b
b
a
b
b
a
b
b
2 mM
5 mM
10 mM
Glutamine concentration
SW480
SW620
LiM2
f
OCR fold change (log2)
0.4
0.3
0.2
0.1
0
-0.1
-0.2
-0.3
a
b
b
a
b
b
100 uM
200 uM
Palmitate concentration
SW480
SW620
LiM2
g
Glucose mito fuel assay % OCR
35
30
25
20
15
10
5
0
-5
-10
-15
ab
b
b
a
b
b
a
b
b
Capacity
Dependency
Flexibility
SW480
SW620
LiM2
h
Glutamine mito fuel assay % OCR
25
20
15
10
5
0
-5
-10
b
b
b
a
b
b
c
ab
b
Capacity
Dependency
Flexibility
SW480
SW620
LiM2
i
Fatty acids mito fuel % OCR
18
16
14
12
10
8
6
4
2
0
a
b
b
a
b
b
a
b
ab
Capacity
Dependency
Flexibility
SW480
SW620
LiM2

Figure S2

Figure S2. Metabolic changes under metastatic progression. Related to Figure 2. (**a**) P-PDH / PDH ratio from the experiment in Fig. 2D. (**b**) GLS and GLUD1 protein level western blotting quantification normalised by TATA box protein levels. (**c**) Oxygen Consumption Rate (OCR) values at a baseline condition of 12.5 mM glucose and 4 mM glutamine. (**d**) Oxygen Consumption Rate (OCR) after glucose titration under a baseline of 4 mM glutamine or (**e**) after glutamine titration under a baseline of 3 mM glucose or (**f**) after palmitate titration with a baseline of 3 mM glucose. (**g**) Mito fuel assay for glucose, glutamine (**h**) and palmitate (i) for each cell line. Percentage of oxygen consumption rate (OCR) with respect to basal OCR in *c*. All parameters were assessed with the injections of 2 µM UK5099, 3 µM BPTES and 4 µM etomoxir. [a,b,c] A one-way ANOVA was performed for the factor "cell line" except for the panel M, which was "condition", and Scheffe's test was used for multiple comparisons. Groups sharing the same letter do not show a significant difference with α=0.05.

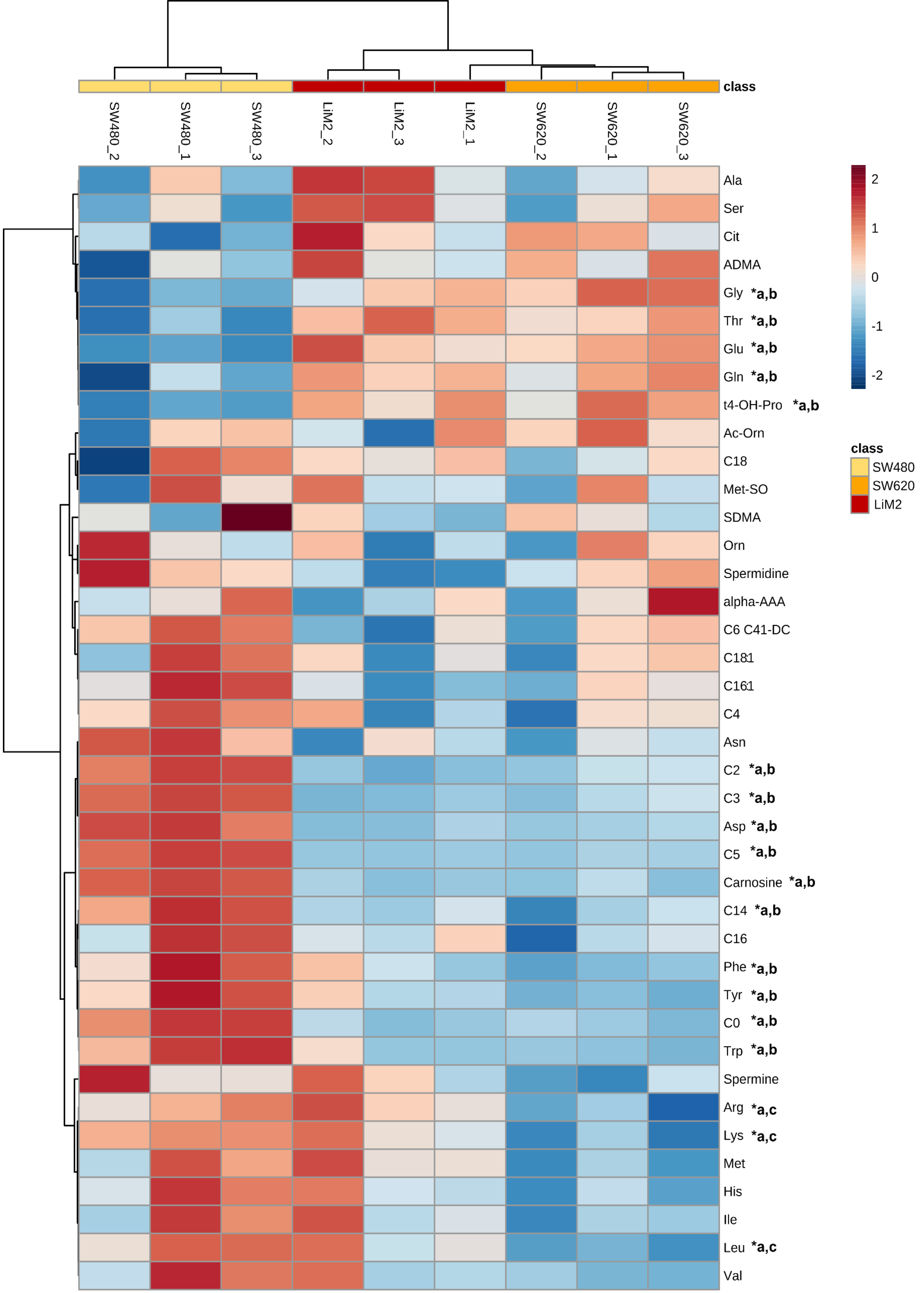

class
SW480_2
SW480_1
SW480_3
LiM2_2
LiM2_3
LiM2_1
SW620_2
SW620_1
SW620_3
Ala
Ser
Cit
ADMA
Gly *a,b
Thr *a,b
Glu *a,b
Gln *a,b
t4-OH-Pro *a,b
Ac-Orn
C18
Met-SO
SDMA
Orn
Spermidine
alpha-AAA
C6 C41-DC
C181
C161
C4
Asn
C2 *a,b
C3 *a,b
Asp *a,b
C5 *a,b
Carnosine *a,b
C14 *a,b
C16
Phe *a,b
Tyr *a,b
C0 *a,b
Trp *a,b
Spermine
Arg *a,c
Lys *a,c
Met
His
Ile
Leu *a,c
Val
2
1
0
-1
-2
class
SW480
SW620
LiM2

Figure S3

Figure S3. Metabolic changes under metastatic progression, intracellular metabolites measurement. Related to Figure 2. Heatmap for intracellular content of amino acid, biogenic amines, and acyl-carnitines, obtained by the Absolute IDQ p180 kit (Biocrates Life Sciences AG) after 24 h incubation with DMEM 12.5 mM Glc and 4 mM Gln, 5% FBS and 1% S/P. Metabolite concentrations were normalised by protein concentration, mean-centred and scaled. Hierarchical clustering for samples and features was performed using the Ward algorithm and using Euclidian distance as a measure of similarity. ANOVA was used to determine statistically significant features, and Fisher's least significant difference method was used to evaluate statistically significant differences (a: SW480-SW620, b: SW480-LiM1, c: SW480-LiM2, d: SW620-LiM1, e: SW620- LiM2, f:LiM1 – LiM2, g: NCM460-SW480, h: NCM460-SW620). C*x:y* denotes an acylcarnitine carrying fatty acids with *x* carbons and *y* unsaturations.

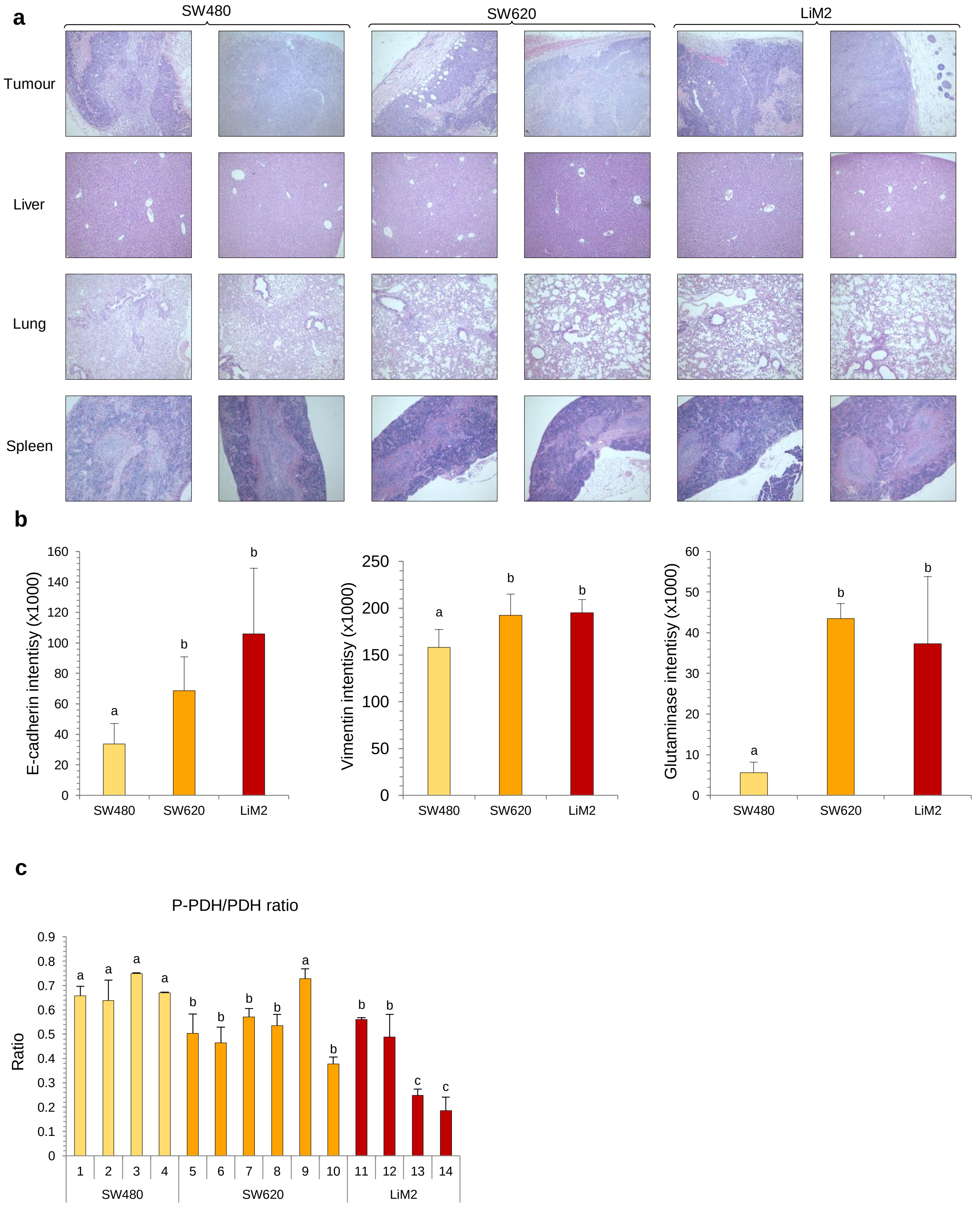

a
SW480
SW620
LiM2
Tumour
Liver
Lung
Spleen
b
E-cadherin intentisy (x1000)
Vimentin intentisy (x1000)
Glutaminase intentisy (x1000)
SW480
SW620
LiM2
c
P-PDH/PDH ratio
Ratio
1
2
3
4
5
6
7
8
9
10
11
12
13
14
SW480
SW620
LiM2

Figure S4

Figure S4. The metabolic adaptation of the metastatic cell lines observed *in vitro* is maintained in an *in vivo* scenario. Related to Figure 3. (**a**) Haematoxylin and eosin staining of paraffin-embedded tissues (tumour, liver, lung, spleen) (40X). (**b**) Quantification of immunohistochemical staining using ImageJ software from the images in Figure 3B. (**c**) P-PDH / PDH ratio from the experiment in Figure 3c. 1 – 14 are extracts from different mice that were injected with SW480 (1-4), SW620 (5-10) or LiM2 (11-14). [a,b,c] A one-way ANOVA was performed for the factor "cell line" in case of panel b, and "mouse" in case of panel c, and Scheffe's test was used for multiple comparisons. Groups sharing the same letter do not show a significant difference with α=0.05.

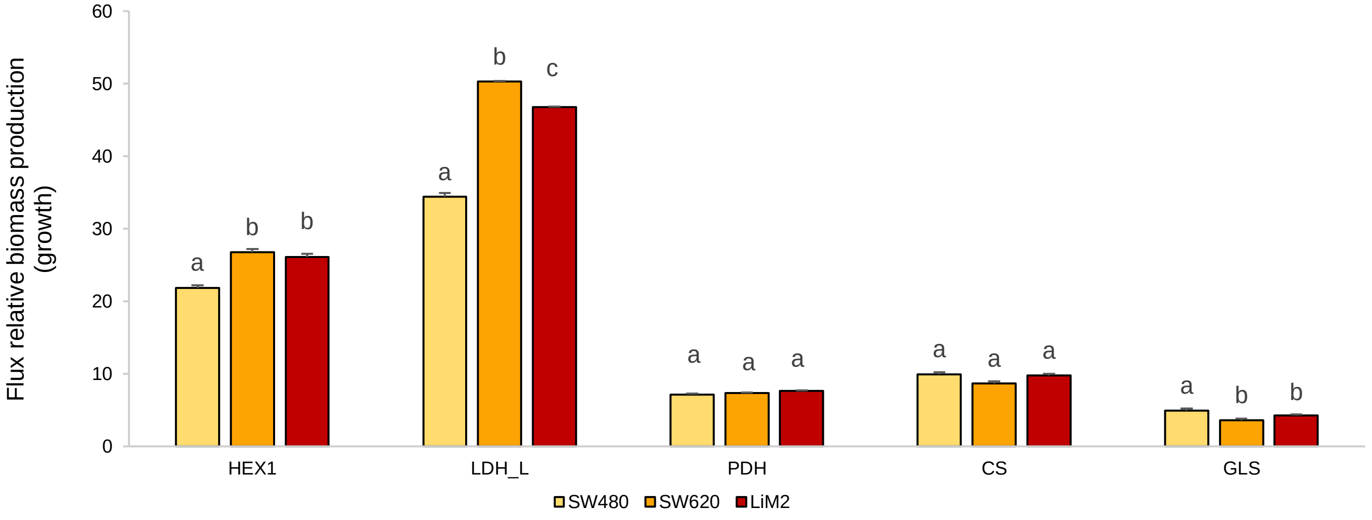

Flux relative biomass production (growth)
60
50
40
30
20
10
0
a
b
b
a
b
c
a
a
a
a
a
a
a
b
b
HEX1
LDH_L
PDH
CS
GLS
SW480
SW620
LiM2

Figure S5

Figure S5. Fluxes relative to biomass production. Related to Figure 4. Fluxes through HEX1 (hexokinase), LDH-L (lactate dehydrogenase), PDH (pyruvate dehydrogenase), CS (citrate synthase) and GLS (glutaminase) relative to the flux of the biomass reaction. The biomass reaction represents the demand of building blocks, ATP and reductive power for growth and proliferation and it's flux is proportional to the experimentally determined growth rate. [a,b,c] denote cell lines with an overlap of the sampled relative flux values for a given reaction.

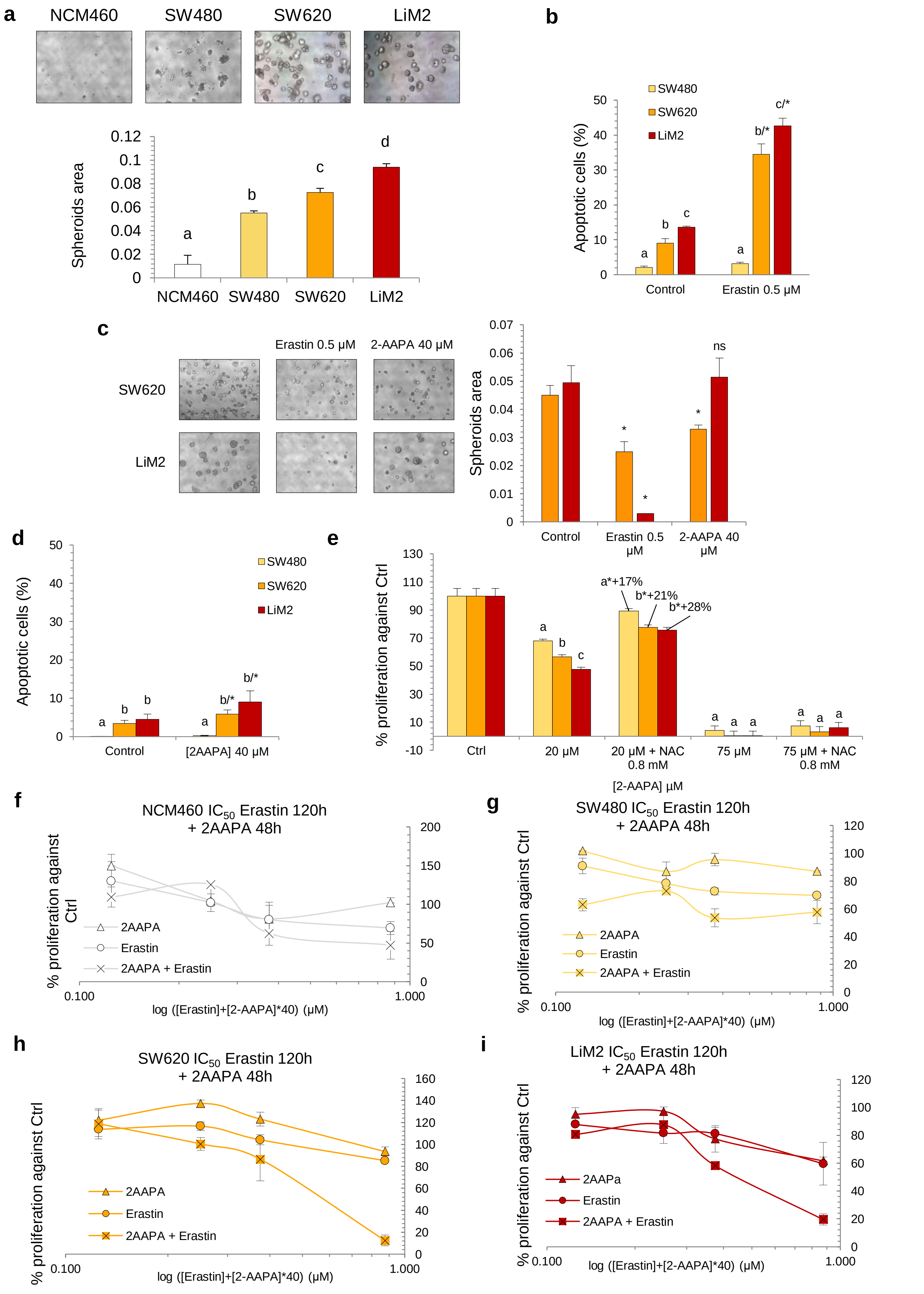

a
NCM460
SW480
SW620
LiM2
Spheroids area
b
SW480
SW620
LiM2
Apoptotic cells (%)
Control
Erastin 0.5 μM
c
Erastin 0.5 μM
2-AAPA 40 μM
SW620
LiM2
Spheroids area
ns
Control
Erastin 0.5 μM
2-AAPA 40 μM
d
Apoptotic cells (%)
Control
[2AAPA] 40 μM
e
% proliferation against Ctrl
a*+17%
b*+21%
b*+28%
Ctrl
20 μM
20 μM + NAC 0.8 mM
75 μM
75 μM + NAC 0.8 mM
[2-AAPA] μM
f
NCM460 IC50 Erastin 120h + 2AAPA 48h
% proliferation against Ctrl
2AAPA
Erastin
2AAPA + Erastin
log ([Erastin]+[2-AAPA]*40) (μM)
g
SW480 IC50 Erastin 120h + 2AAPA 48h
h
SW620 IC50 Erastin 120h + 2AAPA 48h
i
LiM2 IC50 Erastin 120h + 2AAPA 48h
2AAPa

Figure S6

Figure S6. Metastatic cells are dependent on cystine uptake and vulnerable to system xCT and glutathione reductase inhibition. Related to Figure 5. (**a**) Spheroid formation assay for NCM460, SW480, SW620 and LiM2 cell lines. Images of contrast-phase microscope (40X) and quantification of spheroid area using ImageJ software from scanned images. (**b**) Percentage of early apoptotic cells measured by flow cytometry using Annexin V-PI under a concentration of 0.5 μM of erastin or control conditions after 72h incubation. (**c**) Spheroid formation assay adding the inhibitors erastin (0.5 μM) and 2-AAPA (40 μM) for one week. Images of contrast-phase microscope (40X) and quantification of spheroid area using ImageJ software from scanned images.* Student's *t* test for erastin or 2-AAPA vs control conditions, $p<0.05$. (**d**) Percentage of early apoptotic cells measured by flow cytometry using Annexin V-PI under a concentration 40 μM of 2-AAPA or control conditions after 72h incubation. (**e**) Cell proliferation measured by DNA content using HO33342 under control conditions (Ctrl), under 2-AAPA treatments and adding N-acetylcysteine to 2-AAPA treatments (-CYS+NAC). (**f**) – (**i**) IC_{50} curve for erastin alone (120h), 2-AAPA alone (120h) and the combination of both inhibitors (erastin 72h and erastin+2AAPA until 120h) for (**f**) NCM460, (**g**) SW480, (**h**) SW620 and (**i**) LiM2 assessed by cell proliferation measured by DNA content using HO33342. * Student's *t* test for "2-AAPA 20 μM + NAC 0.8 mM" vs "2-AAPA 20 μM" conditions, $p<0.05$. [a,b,c,d] A one-way ANOVA was performed for the factor "cell line", and Scheffe's test was used for multiple comparisons. Groups sharing the same letter do not show a significant difference with α=0.05.

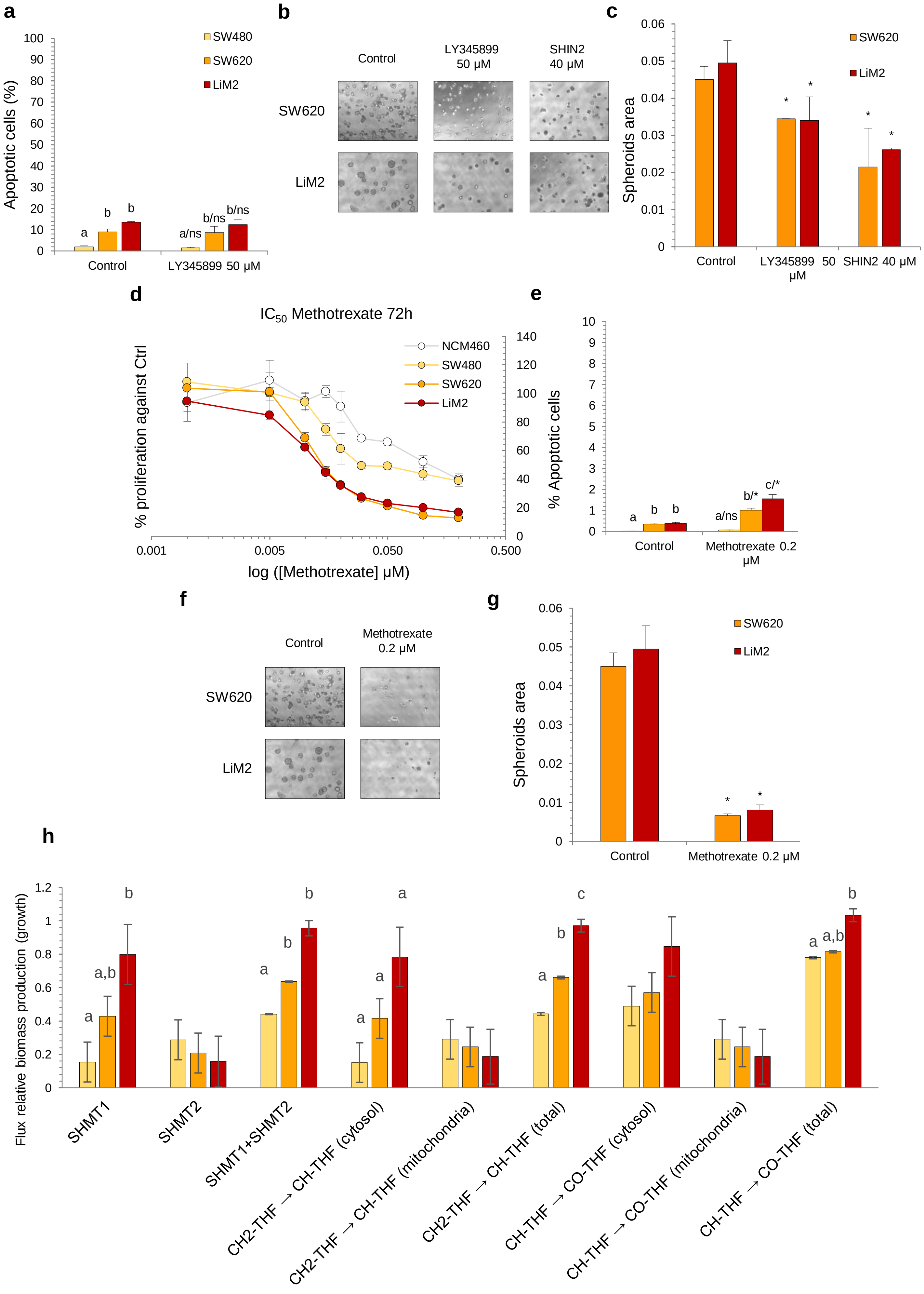

a
Apoptotic cells (%)
SW480
SW620
LiM2
a
b
b
a/ns
b/ns
b/ns
Control
LY345899 50 µM
b
Control
LY345899 50 µM
SHIN2 40 µM
SW620
LiM2
c
Spheroids area
SW620
LiM2
Control
LY345899 50 µM
SHIN2 40 µM
d
IC50 Methotrexate 72h
% proliferation against Ctrl
NCM460
SW480
SW620
LiM2
log ([Methotrexate] µM)
e
% Apoptotic cells
a
b
b
a/ns
b/*
c/*
Control
Methotrexate 0.2 µM
f
Control
Methotrexate 0.2 µM
SW620
LiM2
g
Spheroids area
SW620
LiM2
Control
Methotrexate 0.2 µM
h
Flux relative biomass production (growth)
SHMT1
SHMT2
SHMT1+SHMT2
CH2-THF → CH-THF (cytosol)
CH2-THF → CH-THF (mitochondria)
CH2-THF → CH-THF (total)
CH-THF → CO-THF (cytosol)
CH-THF → CO-THF (mitochondria)
CH-THF → CO-THF (total)

Figure S7

Figure S7. The metastatic cell lines are dependent on the cytosolic branch of folate metabolism. Related to Figure 6. (**a**) Percentage of early apoptotic cells measured by flow cytometry using Annexin V-PI under a concentration of 50 μM of LY345899 or control conditions after 72h incubation. (**b**) and (**c**) Spheroid formation assay adding the inhibitors LY345899 (50 μM) and SHIN2 (40 μM) for one week. (**b**) Images of contrast-phase microscope (40X) and (**c**) quantification of spheroid area using ImageJ software from scanner images. * Student's *t* test for LY345899 or SHIN2 vs control conditions, $p<0.05$. (**d**) Cell viability curve of the DHFR inhibitor methotrexate measured by DNA content using HO33342 after 72h incubation. (**e**) Percentage of apoptotic cells measured by flow cytometry using Annexin V-PI under a concentration of 0.2 μM of methotrexate or control conditions after 72h incubation. (**f**) and (**g**) Spheroid formation assay adding the inhibitor methotrexate (0.2 μM) for one week. (**f**) Images of contrast-phase microscope (40X) and (**g**) quantification of spheroid area using ImageJ software from scanner images. * Student's *t* test for methotrexate vs control conditions, $p<0.05$. [a,b,c] A one-way ANOVA was performed for the factor "cell line", and Scheffe's test was used for multiple comparisons. Groups sharing the same letter do not show a significant difference with α=0.05. **(h)** Predicted flux values for different reactions of cytosolic and mitochondrial folate metabolism relative to the flux through the biomass reaction. [a,b,c] denote cell lines with an overlap of the sampled flux values for a given reaction.

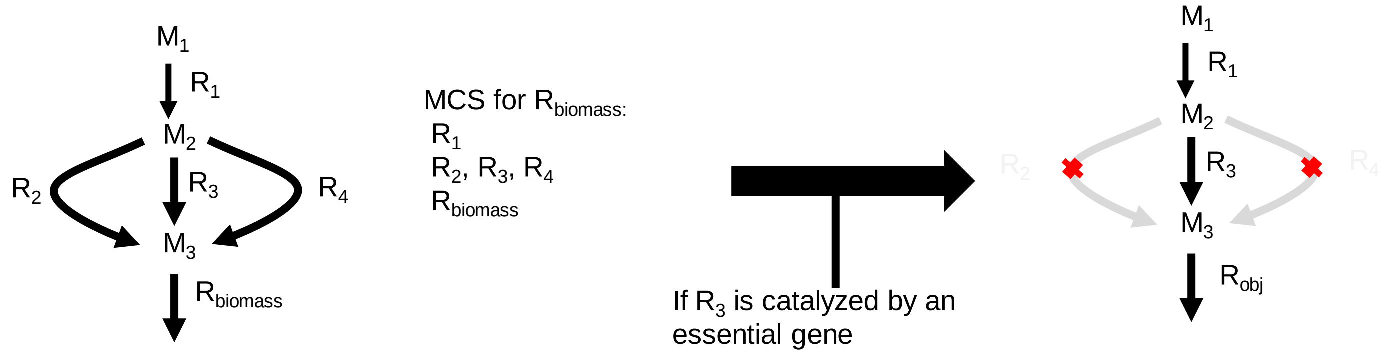
M1
R1
M2
R2
R3
R4
M3
Rbiomass
MCS for Rbiomass:
R1
R2, R3, R4
Rbiomass
If R3 is catalyzed by an essential gene
M1
R1
M2
R2
R3
R4
M3
Robj

Figure S8

Figure S8. Integrating known gene dependencies using MCS analysis. Reactions catalysed by genes described as essential can be assumed to be part of a MCS where the other reactions are already inactive. In this example, because R3 is catalysed by a gene described as essential, R2 and R4 can be assumed to be inactive.

Supplementary Methods

Western blotting

Equal amounts of protein extracts were separated by SDS-PAGE gel (10%) and transferred to polyvinylidene fluoride transfer membranes. Membranes were blocked with 5% non-fat milk in PBS-0.1% Tween 20 and incubated with a specific primary antibody followed by an incubation with the appropriate Horse radish peroxidase (HRP)-labelled secondary antibody. HRP activity was assessed with Immobilon ECL Western Blotting Detection Kit Reagent and detected by exposition with photographic film. Antibodies used in the study included rabbit anti-E-cadherin (ab1416, Abcam, UK) diluted at 1:1000, mouse anti-N-cadherin (610920 BD Transductions Laboratories, USA) diluted at 1:1000, mouse anti-vimentin (MS-129, Thermo Fisher Scientific, USA) diluted at 1:1000, rabbit anti-ZEB2 (AV33694, Sigma-Aldrich, USA) diluted at 1:1000, rabbit anti-Twist ½ (GTX127310, Genetex, USA) diluted at 1:1000, rabbit anti-SNAI1 (GTX125918, Genetex, USA) diluted at 1:1000, rabbit anti-NF-κB (sc-103, Santa Cruz Biotechnology, USA) diluted at 1:2000, rabbit anti-AKT (9272S, Cell Signalling Technology) diluted at 1:500, rabbit anti-P-AKT (9271, Cell Signaling Technology, USA) diluted at 1:1000, rabbit anti-cMyc (Y69) (ab32072, Abcam, UK) diluted at 1:10000, mouse anti-PDH (sub E1) (ab110330, Abcam, UK) diluted at 1:1000, rabbit anti-P-PDH (sub E1-A) (S293) (ABS204, Merck Millipore, USA) diluted at 1:10000, rabbit anti-PC (ab126707, Abcam, UK) diluted at 1:10000), rabbit anti-GLUD1 (ab166618, Abcam, UK) diluted at 1:1000, rabbit anti-GLS (ab93434, Abcam, UK) diluted at 1:1000 and rabbit anti-TATA (TBS) (ab63766, Abcam, UK) diluted at 1:1000. The TATA antibody was used as a loading control. Secondary antibodies included Anti-Mouse (GR304350-1, Abcam, UK) diluted at 1:20000 and Anti-Rabbit (GR297013-4, Abcam, UK) diluted at 1:20000 and Anti-Goat (sc-2020, Santa Cruz Biotechnology, USA) diluted at 1:10000.

Immunohistochemistry

After fixation in 4% paraformaldehyde the tissues were paraffin-embedded and slides were obtained. Then, deparaffination was achieved by washes with xylene and decreasing ethanol concentrations until rehydration of the slides. Then, antigen unmasking was performed in 95-100ºC bath for 20 minutes with high pH retrieval solution (from Dako kit EnVision dual Link System-HRP DAB+ K4065, Agilent Technologies, USA). Then, the slides were blocked with the Dual Endogenous Enzyme Block solution from the kit, washed with the wash buffer from the kit and incubated O/N at 4ºC in a humidified chamber with the corresponding antibodies (rabbit anti-GLS (ab93434, Abcam, UK) diluted at 1:100 with antibody diluent solution, rabbit anti-E-cadherin (GTX629691, Genetex, USA) diluted at 1:500, rabbit anti-Vimentin (GTX629744, Genetex, USA) diluted at 1:500). After washing, the slides were incubated with the labelled polymer (-HRP) from the kit and antibody binding was stained by the DAB+ chromogen solution. The slides were dehydrated and mounted with Dako Mounting Medium CS703.

Spectrophotometric measurements

Determination of glucose was performed using the Hexokinase and D-glucose-6-phosphate Dehydrogenase reactions (ABX Pentra Glucose HK CP, Horiba ABX, Japan) and NADPH release was measured at 340 nm in a COBAS Mira Plus spectrophotometer (Horiba ABX, Japan). Lactate was measured using Lactate Dehydrogenase at 87.7 U/mL, 1.55 mg/mL NAD^+ in 0.2 M hydrazine, 12 mM EDTA pH 9 buffer and NADH release was measured at 340nm. Glutamate was quantified by glutamate dehydrogenase at 39 U/mL, 2.41 mM ADP, 3.9 mM NAD^+ in 0.5 M hydrazine, 0.5 M glycine pH 9 buffer and NADH release was measured at 340 nm. Glutamine was measured indirectly by first transforming it to glutamate by glutaminase in 125 mM acetate pH 5 buffer for 30 minutes at 37ºC and then performing the same reaction as for glutamate concentration determination.

OCR measurements, Mito Stress and Mito Fuel Assays

Plating technique involved 100 μL seeding of cell suspension and 100 μL extra addition of medium 3 hours later once cells had attached to the surface. After overnight growth, medium was replaced with Seahorse medium (buffer-free DMEM supplemented with glucose, glutamine and antibiotics). The plates were equilibrated in a 37ºC-incubator without CO_2 for 60 minutes. The cartridge with the sensors was hydrated with calibration solution (Seahorse Bioscience, USA) overnight at 37ºC and loaded into the Seahorse Analyser at least 30 minutes before starting the experiment to calibrate the sensors. For glucose, glutamine and fatty acids Mito-Fuel Assay, baseline conditions were used (12.5 mM glucose, 4 mM glutamine and 1% streptomycin and penicillin). The capacity, dependency and flexibility for each substrate were assessed with the injections of 2 μM UK5099 (inhibitor of pyruvate carrier) 3 μM BPTES (inhibitor of glutaminase) and 4 μM etomoxir (inhibitor of CPT1A).

Targeted metabolomics

For the quantification of intracellular metabolites, 5 x 10^6 cells in 100 mm plates (SW480, SW620 and LiM2 cell lines) were trypsinised and centrifuged at 500g for 5 minutes and metabolites were extracted from cell pellets. Pellet was resuspended in 70 μL of 85:15 EtOH:PBS buffer and sonicated 3 times for 5 seconds each, then submerged in liquid nitrogen for 30 seconds and thawed at 95ºC. Then, it was centrifuged 20,000 g, 5 minutes at 4ºC, supernatant was collected, and protein content was measured. For the determination of metabolites uptake and production, metabolites were extracted from cell media taken from the beginning and the end of a 24 h incubation in exponential growth and high confluence conditions and cell number was determined. Both extracts from pellets and media were plated in the Biocrates plate together with the calibration standards, and derivatised to be ready for UHPLC-MS reading (for amino acids and biogenic amines) and FIA-MS/MS (for lipids, sugars and acylcarnitines) according to the manufacturer's instructions. A total of 21 amino acids, 19 biogenic amines, 90 glycerophospholipids, 15 sphingolipids, 40 acylcarnitines and hexose sugars were analysed.

Stable-isotope resolved metabolomics in vitro

For polar intracellular metabolites analysis, the cells were washed with ice-cold PBS and scrapped with 1:1 metanol:water (adding first 1 mL of methanol, waiting for 1 minute and adding 1 mL of milliQ water afterwards). Then, the samples were sonicated (3 cycles of 5 seconds) and 2 mL of cold chloroform were added. After gentle shaking (30 minutes at 4ºC) the samples were centrifuged (20000 g, 15 minutes at 4ºC) and supernatant was completely dried under air flow. The extracted metabolites were derivatised by adding 50 μL of 2% methoxamine hydrochloride in pyridine for 90 minutes at 37ºC and (N-methyl-N-tert-butyldimethylsilyl) trifluoroacetamide + 1% tertbutyldimethylchlorosilate) for 60 minutes at 55ºC before GC-MS analysis (Agilent 7890A gas chromatograph coupled to a Agilent 5975C mass spectrometer, Agilent Technologies, Santa Clara, CA, USA) using electron impact mode.

For intracellular ribose analysis, RNA was isolated from cell pellets using Trizol reagent, mixing it with chloroform. The aqueous phase was obtained, and cold isopropanol was added and centrifuged at 12000 g, 15 minutes at 4ºC. The samples were washed several times using cold 75% ethanol and isolated RNA was quantified using a Nanodrop spectrophotometer (ND 1000 V3.1.0, Thermo Fisher Scientific). The samples of purified RNA were hydrolysed in 2 mL of 2 M HCl at 100ºC for 2 hours, dried under air flow and derivatised using 100 μL of 2% hydroxylamine hydrochloride in pyridine at 100ºC for 30 minutes and 75 μL of acetic anhydride at 100ºC for 1 hour. Then, samples were dried under N_2 flow and resuspended in ethyl acetate before GC-MS analysis using chemical ionisation mode.

For extracellular glucose analysis, glucose from cell culture media was isolated using Dowex-1X8/Dowex-50WX8 ion-exchange columns and samples were dried under air flow.

Purified glucose was derivatised by incubation with 100 μL of 2% hydroxylamine hydrochloride in pyridine at 100ºC for 30 minutes and 75 μL of acetic anhydride at 100ºC for 1 hour. Then, samples were dried under N_2 flow and resuspended in ethyl acetate before GC-MS analysis using chemical ionisation mode.

For extracellular lactate analysis, lactate from cell culture media was isolated adding HCl and 1 mL of ethyl acetate previous to air flow drying. Derivatisation was performed by incubation with 200 μL of 2,2-dimethoxypropane and 50 μL of 0.5 N methanolic HCl at 75ºC for 1 hour and then adding 60 μL of n-propylamine at 100ºC for 1 additional hour. After drying under N_2 flow, samples were filtered using glass wool through a Pasteur pipette and dried again under N_2 flow. Then, samples were resuspended and incubated with 200 μL of dichloromethane and 15 μL of heptafluorobutyric anhydride at room temperature for 10 minutes, dried under N_2 and resuspended under ethyl acetate before GC-MS analysis under chemical ionisation mode.

Isotopologue (mass isotopomer) fractions for each metabolite were measured from raw data using MSD5975C Data Analysis (Agilent Technologies), integrating peak areas and then correcting for natural heavy isotope enrichment using the Midcor software package [1].

Stable-isotope resolved metabolomics in vivo

Metabolites were extracted from 10 mg of dry tissue with methanol:chloroform 1:2. First, 600 μL of cold methanol containing 5 nmols of scyllo-inositol, 10 nmols of N-norleucine and 1.5 nmols of $^{15}N^{13}C$-valine were added as internal standards. Then, 1200 μL of chloroform was added and after extensive vortexing, the tubes were sonicated (3 times, 8 minutes, 4ºC) and centrifugated (20 minutes, 4ºC, 20000g). The supernatant was dried in speed-vacuum machine and 500 μL of methanol:water was added to the pellet for a second extraction following the same steps as before, adding it to the first dried supernatant and drying it as well. Separation of polar and apolar phase was performed by addition of 3:3:1 methanol:water:chloroform (150:150:50 μL) and vortexing and centrifugating (30 minutes, 4ºC , 20000g). The extracted metabolites in the polar phase were derivatised by adding 50 μL of 2% methoxamine hydrochloride in pyridine for 90 minutes at 37ºC and (N-methyl-N-tert-butyldimethylsilyl) trifluoroacetamide + 1% tertbutyldimethylchlorosilate) for 60 minutes at 55ºC before GC-MS analysis (Agilent 7890A gas chromatograph coupled to a Agilent 7890A mass spectrometer, Agilent Technologies, Santa Clara, CA, USA) using electron impact mode.

Statistical analyses

All experiments were performed at least in triplicates and repeated two or more times. Statistical analyses of experimental measures were performed using the Agricolae package for R. More in detail, for comparisons that are between the cell lines (SW480 – SW620 – LiM2) we used a one-way ANOVA for the factor "cell line", and Scheffe's test for multiple comparisons. Groups that are indicated with the same letter are not significantly different ($p>0.05$). For comparisons between two conditions (e.g., before and after drug administration), we used Student's t test with $p<0.05$, and we used asterisks to indicate significant differences. We used the Shapiro-Wilk test to assess normal distribution of the experimental data and Dixon's Q-test to identify outliers and Levene's test to test homogeneity of variances. Differential gene expression between cell lines in the batch corrected gene expression data was determined using the Linear Models for Microarray Data (limma) package for R, defining "cell line" as a factor [2]. Targeted intracellular metabolomics data was analysed using the Metaboanlyst web server [3]. Concentrations were normalised by protein and transformed into a Log2 scale. Significant features were determined using a one-way (ANOVA) with a cut-off of 0.05 false discovery rate (FDR). This was followed by a post-hoc analyses using Fisher's least significant difference method (Fisher's LSD) to identify potential significant differences between cell line pairs. To build a heatmap, metabolite concentrations were mean-centred and scaled; Hierarchical clustering for samples and features was performed using the Ward algorithm and Euclidian distance as measure of similarity. The

Combination Index for drug combinations, was computed with the CompuSyn software (ComboSyn, Inc., USA) as described by Chou and Talalay [4]. With that software, CI<1, CI=1 and CI>1 indicates synergy, additive effect and antagonism, respectively.

Multiomics data integration

Integrating extracellular flux measurements

Measures of extracellular metabolite concentrations (measured either through spectrophotometric methods or targeted metabolomics) were used to compute the rate of metabolite uptake or secretion normalised by cellular volume using the following equation [5]:

$$v_{M_{ex}} = \frac{M_1 - M_0}{N_1 - N_0} \cdot \mu \cdot \frac{1}{vol} \qquad (1)$$

Where,
$v_{M_{ex}}$ is the estimated rate at which the metabolite M is produced/consumed per cell volume (μmol·h^{-1}·(μl cell volume)$^{-1}$)
M_1 and M_0 are metabolite abundances (μmol) measured at time points t_1 and t_0, respectively;
μ is the growth rate (h^{-1})
N_1 and N_0 are the cell numbers measured at time points t_1 and t_0, respectively.
vol is the cellular volume per cell (μl·$cell^{-1}$).
Exchange reactions in the cell line specific metabolic models were constrained to the 99.5% confidence intervals for $v_{M_{ex}}$ of the corresponding metabolites.

Integrating OCR measurements, Mito Stress and Mito Fuel Assays

Seahorse measurements were integrated to cell line specific metabolic models to constraint the oxidative metabolism. Firstly, OCR measurements were used to constrain the rate of oxygen consumption in the cell line specific models. Next, the percentage of OCR associated with ATP synthase, measured in the Mito Stress Assay, was used to constrain the flux through ATP synthase. Finally, from the Mito Fuel Assay, the dependency and capacity for fatty acid oxidation were used to define the lower and upper bound, respectively, for the transport of palmitate into the mitochondria.

Integrating intracellular amino acid and biogenic amines measurements

Metabolomics measured in the cellular pellet were integrated with the proliferation rate to account for the dilution associated with proliferation. This was represented by adding a sink reaction to measured metabolites that represented the requirements of metabolite synthesis to maintain the concentrations of such metabolite in steady state[6].

$$v_{M_{sink}} = [M] \cdot p \cdot \mu \qquad (2)$$

Where:
$[M]$ is the concentration of metabolite M (μmol/mg prot)
p is the protein per cellular volume (mg prot/μl cell volume)
μ: is the proliferation rate in h^{-1}
$v_{M_{sink}}$ is the estimated flux through the sink reaction

To account for uncertainty, the 99.5% confidence intervals for $v_{M_{sink}}$ were used as flux boundaries in the model.

Using lipidomic measurements to personalise the biomass function

Across the analysed conditions, the most abundant phospholipids were the phosphatidylcholines (PC aa) PC aa C34:1, PC aa C34:2, PC aa C36:1 and PC aa C36:2 (Table S5). Such species represent isomeric phosphatidylcholines C x:y where x is the total carbon number of both chains and y is the total number of unsaturations. The relative abundance of such species in each cell line was used to customise the biomass function in each cell line specific model.

Due to the large number of potential fatty acid chain combinations, Recon 2 [7] does not simulate individual phospholipid species. Instead, it simulates the fatty acid chains in phospholipids through an artificial "Rtotalcoa" metabolite that is synthesised from a combination of acyls-CoA with stoichiometric coefficients representing the relative abundance of each fatty acid chain in phospholipids.

The most abundant fatty acids in mammals are reported to be oleic (C18:1), palmitate (C16:0), stearic acid (18:0) and palmitoleic (16:1), in that order [8] (Table S5). Hence, we assumed that the side chains of the phosphatidylcholines primarily consisted of such fatty acids and their relative abundance was used as coefficients for "Rtotalcoa" synthesis (Table S6).

It is worth noting, that even if other combinations of acyl-CoA can give rise to the measured isobaric phosphatidylcholines, the metabolic cost (i.e., NADPH, ATP and Acetyl-CoA) of producing any given phosphatidylcholine will depend primarily on the total length of the fatty acid chains and the number of unsaturations. Hence, the assumption that oleic, palmitate, stearic acid and palmitoleic, are the components of "Rtotalcoa" it is a valid approximation.

Integrating growth rates

The proliferation rates for each cell line were integrated to reflect the different proliferation rates of the cell lines of study. First maximum biomass production in SW620($v_{biomass}^{SW620}$) was computed using flux balance analysis (FBA) [9]. Then the biomass production of the remaining cell lines was set as follows:

$$v_{biomass}^{cell\ line} = v_{biomass}^{SW620} \cdot \frac{\mu^{cell\ line}}{\mu^{SW620}} \qquad (3)$$

$v_{biomass}^{cell\ line}$ was then set as the upper bound for the biomass reaction allowing the models to accurately reflect the different growth rates of the different cell lines.

^{13}C MFA and GSMM integration

The central metabolism flux maps consistent with the measured ^{13}C propagation and the measured rates of uptake and secretion for glucose, lactate and amino acids, and respiration data were computed in the framework of ^{13}C MFA [10].

^{13}C MFA was performed in a metabolic network of central carbon metabolism built from Recon2 [7]. The network comprised 347 reactions including, glycolysis, TCA cycle, pentose phosphate pathway, energy and redox metabolism and the main pathways of amino acid metabolism and biomass components synthesis.

Using the software INCA (isotopomer network compartmental analysis) [11], 95% confidence intervals for flux values were computed for all reactions in the network. Such confidence intervals were added as flux boundaries in the cell line specific GSMMs. While most reactions could be directly mapped, some reactions that were defined as single reactions in the ^{13}C MFA network were defined as multiple reactions in Recon 2 (e.g., a reaction that can occur in multiple compartments or a reaction that can take either NAD or NAPD as a cofactor). In such instances, the ^{13}C MFA confidence intervals were used to constraint the summation of the flux through the equivalent reactions in Recon 2.

Transcriptomics

Transcriptomics data were obtained from the gene expression omnibus repository [12]. Data was taken from GSE1323 (SW480-SW620) [13] and GSE33350 (SW620-LIM1-LIM2) [14]. Batch effect was corrected using the sva package for R[15].

Transcriptomics were mapped to reactions using the gene protein reaction rules (GPR) defined in Recon 2 [7]. In detail, first OR operators were replaced by "MAX()" operators and AND operators by "MIN()" in the GPR expressions. Then, GPR expressions for each reaction were evaluated, replacing gene IDs by their respective transcript abundances. Under such a system, a reaction catalysed by multiple isoenzymes will be mapped to the transcript abundance of the most expressed isoenzymes, while reactions catalysed by protein complexes will be mapped to the leas abundant transcript of the complex's components

Minimal cut set analysis

From the project DRIVE database, the list of essential metabolic genes in SW480 and SW620 was obtained [16]. A gene was considered essential if it's Redundant siRNA Activity (RSA)[17] score was equal or lower than -2. Conversely, a gene was defined as dispensable (i.e., not essential) if it had an RSA score larger or equal than -1.

Minimal cut set (MCS) analysis [18,19] was performed to identify the MCSs containing the essential genes identified in Project DRIVE. MCS are minimal sets of genes or reactions whose simultaneous removal directly blocks a metabolic task, biomass production in this case. MCS analysis was run in Recon 2 [7] assuming an extracellular metabolite availability in the media defined from the composition of DMEM. Furthermore, blocked reactions were removed and reactions that could only be part of linear pathways were grouped. MCS analysis was set to seek 8 MCS containing each Project DRIVE essential gene and all reactions were evaluated as possible gene set candidates. As reported by Apaolaza *et a*l. [18], in some instances, the MCS algorithm can fail to reach optimality and provide a gene set, that although it contains MCS, is not minimal. When this occurred, we used FASTL[20] to identify the MCS within the returned set.

It can be assumed that if a gene metabolic function is essential in a given cell line, then this gene will be part of an MCS where the other MCS members have low activity. To integrate this information the following workflow was used for each cell line:

- *Rank essential genes based on their RSA score (low to high)*
- *For each essential gene in the ranked list:*
    - *Rank each MCS containing the gene of interest based on the total gene expression evidence of the reactions in the set, excluding reactions associated with the gene of interest. This is achieved by mapping transcriptomics to reactions using the GPR rules defined in Recon 2 and adding the gene expression value associated with each reaction in the MCS. MCS with more than 8 reactions, not counting reactions associated with the gene of interest, were excluded.*
    - *For each MCS in the ranked list:*
        - *Implement the MCS. Force the reactions in the set to be inactive, excluding the reactions associated with the essential gene. Following the definition of MCS, the gene of interest is now an essential gene.*
        - *Use flux variability analysis* [21] *to evaluate if the MCS is consistent with:*
            - *Intracellular metabolomics (i.e., all detected metabolites can be produced).*
            - *All measured rates of uptake and secretion and OCR measurements.*
            - *^{13}C MFA flux intervals.*
        - *Systematically simulate the effect of gene KO for all metabolic genes defined as dispensable in Project DRIVE using FBA. Compute the number of false positives (dispensable genes in Project DRIVE that are predicted as essential by the model).*
        - *If implementing the MCS a) increases the number of false positives or b) is inconsistent with metabolomics, flux measurements or ^{13}C MFA:*
            - *Revert the MCS implementation.*
            - *Continue with the next MCS in the ranked list.*
        - *Else:*
            - *Continue to the next essential gene.*

For LiM2, we implemented the MCSs 1) shared between SW480 and SW620 and 2) the MCSs in SW620 where none of the genes associated with the reactions in the MCS were significantly overexpressed in LiM2.

In total, 8 ,7 and 6 MCSs were implemented into SW480, SW620 and LiM2, respectively.

Gene Inactivation Moderated by Metabolism, and Expression (GIMME)

GIMME[22,23] was used to integrate transcriptomics together with the aforementioned data sets (e.g., extracellular fluxes, metabolomics, ^{13}C MFA and MCS) to build cell line specific genome-scale flux maps. GIMME optimises biomass production and then performs a second optimisation where fluxes through reactions are minimised with a weight that is a function of the gene expression value mapped to each reaction. In our analysis, the minimisation weight (w_i) of each reaction was defined as follows:

$$w_i = 1 + ge_{max} - ge_i \qquad (4)$$

where,
ge_i is the gene expression value mapped to reaction *i following the* GPR rules defined in Recon2.
ge_{max} is the maximum gene expression value in the set of all metabolic genes.

For each cell line, GIMME was run in a condition-specific GSMM obtained by implementing MCS, growth rates, the personalised biomass function, metabolomics, respiration parameters, rates of metabolite uptake and secretion, ^{13}C MFA in Recon2 as detailed in the previous sections. From the optimal GIMME solution, any inactive reaction with a mapped gene expression value under the 25th percentile of metabolic genes expression was removed. This allows pruning reactions catalysed by lowly expressed enzymes from the network. Next, Following the GIM^{3}E approach [23], each flux was maximised and minimised to identify the ranges of feasible fluxes within the optimal GIMME solution with a tolerance of 0.1%. This space of solution represents the space of most likely flux distributions in the conditions of study.

Finally, GIMME was also run with 10% tolerance in a model integrating cell line specific MCS and biomass function but no other cell lines specific measurements such as ^{13}C MFA or metabolomics. Rather than representing the flux map under the conditions of study, the purpose of this model is to represent the metabolic potential of each cell line. Thus, in such model, the flux boundaries for each reaction were modified to always include 0 (i.e., no reaction is forced to be active). These models, that we termed "base model", serve as a framework to simulate gene KO with MOMA [24].

Flux sampling and reference flux distribution selection

From the GIMME algorithm, a space of solutions was identified for each cell line. However, such space was still relatively wide and a strategy to select the most representative and accurate flux distributions from such space was applied:

1. Compute 1000 flux samples from within the GIMME solution space. Flux samples were computed using the Artificially Centered hit-and-run (ACHR) algorithm implemented into COBRApy [25,26]. ACHR was run with a thinning factor of 10000. The thinning factor defines the number of iterations between each returned sample and a large thinning factor reduces the correlation between samples resulting in a more representative set of samples.
2. Using each flux sample as a wild type flux distribution, systematically simulate the KO with MOMA[27] of all metabolic genes analysed in Project DRIVE. Such simulations were run in the cell line specific base model.
3. Use the following equation to give a discrepancy score to each flux sample based on how well they encapsulate available gene essentiality/dispensability data:

$$S_j = \sum_{i \in DRIVE} max(2 + RSA_i\ ,0) \cdot \left(1 - \frac{BiomassKO_i}{Biomass_{WT}}\right) \qquad (5)$$

Where:
S_j is the discrepancy score for flux sample j
RSA_i is the RSA score for gene i (RSA scores have negative values and more negative values indicate more dependency on a gene's function). For LiM2, the DRIVE RSA measurements for SW620 were used as it was the most closely related cell line.
$BiomassKO_i$ is the flux through the biomass reaction when the KO of gene i is simulated with MOMA [27] using flux sample j as input.
$Biomass_{WT}$ is the flux through the biomass reaction in the wild type (i.e., with no reaction inactivated).

4. Select the top 100 flux samples with the least discrepancy score. The average of such flux samples will be used as reference flux distribution for the cell line of study.

Such an approach primarily serves to reduce the number of false positives (i.e., genes predicted as essential by the model and described as dispensable in DRIVE) that might emerge from unrepresentative flux distributions.

References

1. Selivanov, V.A.; Benito, A.A.; Miranda, A.; Aguilar, E.; Polat, I.H.; Centelles, J.J.; Jayaraman, A.; Lee, P.W.N.N.; Marin, S.; Cascante, M. MIDcor, an R-program for deciphering mass interferences in mass spectra of metabolites enriched in stable isotopes. *BMC Bioinformatics* **2017**, *18*, 88, doi:10.1186/s12859-017-1513-3.

2. Ritchie, M.E.; Phipson, B.; Wu, D.; Hu, Y.; Law, C.W.; Shi, W.; Smyth, G.K. limma powers differential expression analyses for RNA-sequencing and microarray studies. *Nucleic Acids Res.* **2015**, *43*, e47, doi:10.1093/nar/gkv007.

3. Chong, J.; Yamamoto, M.; Xia, J. MetaboAnalystR 2.0: From Raw Spectra to Biological Insights. *Metabolites* **2019**, *9*, 57, doi:10.3390/metabo9030057.

4. Chou, T.-C. Drug combination studies and their synergy quantification using the Chou-Talalay method. *Cancer Res.* **2010**, *70*, 440–6, doi:10.1158/0008-5472.CAN-09-1947.

5. Tarrado-Castellarnau, M.; de Atauri, P.; Tarragó-Celada, J.; Perarnau, J.; Yuneva, M.; Thomson, T.M.; Cascante, M. De novo MYC addiction as an adaptive response of cancer cells to CDK4/6 inhibition. *Mol. Syst. Biol.* **2017**, *13*, 940, doi:10.15252/msb.20167321.

6. Reimers, A.-M.; Reimers, A.C. The steady-state assumption in oscillating and growing systems. *J. Theor. Biol.* **2016**, *406*, 176–86, doi:10.1016/j.jtbi.2016.06.031.

7. Swainston, N.; Smallbone, K.; Hefzi, H.; Dobson, P.D.; Brewer, J.; Hanscho, M.; Zielinski, D.C.; Ang, K.S.; Gardiner, N.J.; Gutierrez, J.M.; et al. Recon 2.2: from reconstruction to model of human metabolism. *Metabolomics* **2016**, *12*, 109, doi:10.1007/s11306-016-1051-4.

8. Sheikh, K.; Förster, J.; Nielsen, L.K. Modeling hybridoma cell metabolism using a generic genome-scale metabolic model of Mus musculus. *Biotechnol. Prog.* **2005**, *21*, 112–21, doi:10.1021/bp0498138.

9. Orth, J.D.; Thiele, I.; Palsson, B.Ø. What is flux balance analysis? *Nat. Biotechnol.* **2010**, *28*, 245–8, doi:10.1038/nbt.1614.

10. Antoniewicz, M.R. A guide to 13C metabolic flux analysis for the cancer biologist. *Exp. Mol. Med.* **2018**, *50*, 19, doi:10.1038/s12276-018-0060-y.

11. Young, J.D. INCA: a computational platform for isotopically non-stationary metabolic flux analysis. *Bioinformatics* **2014**, *30*, 1333–5, doi:10.1093/bioinformatics/btu015.

12. Barrett, T.; Wilhite, S.E.; Ledoux, P.; Evangelista, C.; Kim, I.F.; Tomashevsky, M.; Marshall, K.A.; Phillippy, K.H.; Sherman, P.M.; Holko, M.; et al. NCBI GEO: archive for functional genomics data sets--update. *Nucleic Acids Res.* **2013**, *41*, D991-5, doi:10.1093/nar/gks1193.

13. Provenzani, A.; Fronza, R.; Loreni, F.; Pascale, A.; Amadio, M.; Quattrone, A. Global alterations in mRNA polysomal recruitment in a cell model of colorectal cancer progression to metastasis. *Carcinogenesis* **2006**, *27*, 1323–33, doi:10.1093/carcin/bgi377.

14. Urosevic, J.; Garcia-Albéniz, X.; Planet, E.; Real, S.; Céspedes, M.V.; Guiu, M.; Fernandez, E.; Bellmunt, A.; Gawrzak, S.; Pavlovic, M.; et al. Colon cancer cells colonize the lung from established liver metastases through p38 MAPK signalling and PTHLH. *Nat. Cell Biol.* **2014**, *16*, 685–94, doi:10.1038/ncb2977.

15. Leek, J.T.; Johnson, W.E.; Parker, H.S.; Jaffe, A.E.; Storey, J.D. The sva package for removing batch effects and other unwanted variation in high-throughput experiments. *Bioinformatics* **2012**, *28*, 882–3, doi:10.1093/bioinformatics/bts034.

16. McDonald, E.R.; de Weck, A.; Schlabach, M.R.; Billy, E.; Mavrakis, K.J.; Hoffman, G.R.; Belur, D.; Castelletti, D.; Frias, E.; Gampa, K.; et al. Project DRIVE: A Compendium of Cancer Dependencies and Synthetic Lethal Relationships Uncovered by Large-Scale, Deep RNAi Screening. *Cell* **2017**, *170*, 577-592.e10, doi:10.1016/j.cell.2017.07.005.

17. König, R.; Chiang, C.; Tu, B.P.; Yan, S.F.; DeJesus, P.D.; Romero, A.; Bergauer, T.; Orth, A.; Krueger, U.; Zhou, Y.; et al. A probability-based approach for the analysis of large-scale RNAi screens. *Nat. Methods* **2007**, *4*, 847–849, doi:10.1038/nmeth1089.

18. Apaolaza, I.; San José-Eneriz, E.; Tobalina, L.; Miranda, E.; Garate, L.; Agirre, X.; Prósper, F.; Planes, F.J. An in-silico approach to predict and exploit synthetic lethality in cancer metabolism. *Nat. Commun.* **2017**, *8*, 459, doi:10.1038/s41467-017-00555-y.

19. Apaolaza, I.; Valcarcel, L.V.; Planes, F.J. gMCS: fast computation of genetic minimal cut sets in large networks. *Bioinformatics* **2018**, 1–3, doi:10.1093/bioinformatics/bty656.

20. Pratapa, A.; Balachandran, S.; Raman, K. Fast-SL: an efficient algorithm to identify synthetic lethal sets in metabolic networks. *Bioinformatics* **2015**, *31*, 3299–305, doi:10.1093/bioinformatics/btv352.

21. Gudmundsson, S.; Thiele, I. Computationally efficient flux variability analysis. *BMC Bioinformatics* **2010**, *11*, 489, doi:10.1186/1471-2105-11-489.

22. Becker, S.A.; Palsson, B.O. Context-Specific Metabolic Networks Are Consistent with Experiments. *PLoS Comput. Biol.* **2008**, *4*, e1000082, doi:10.1371/journal.pcbi.1000082.

23. Schmidt, B.J.; Ebrahim, A.; Metz, T.O.; Adkins, J.N.; Palsson, B.Ø.; Hyduke, D.R. GIM3E: condition-specific models of cellular metabolism developed from metabolomics and expression data. *Bioinformatics* **2013**, *29*, 2900–8, doi:10.1093/bioinformatics/btt493.

24. Segre, D.; Vitkup, D.; Church, G.M. Analysis of optimality in natural and perturbed metabolic networks. *Proc. Natl. Acad. Sci.* **2002**, *99*, 15112–15117,

doi:10.1073/pnas.232349399.

25. Ebrahim, A.; Lerman, J.A.; Palsson, B.O.; Hyduke, D.R. COBRApy: COnstraints-Based Reconstruction and Analysis for Python. *BMC Syst. Biol.* **2013**, *7*, 74, doi:10.1186/1752-0509-7-74.

26. Heirendt, L.; Arreckx, S.; Pfau, T.; Mendoza, S.N.; Richelle, A.; Heinken, A.; Haraldsdóttir, H.S.; Wachowiak, J.; Keating, S.M.; Vlasov, V.; et al. Creation and analysis of biochemical constraint-based models using the COBRA Toolbox v.3.0. *Nat. Protoc.* **2019**, *14*, 639–702, doi:10.1038/s41596-018-0098-2.

27. Segrè, D.; Vitkup, D.; Church, G.M. Analysis of optimality in natural and perturbed metabolic networks. *Proc. Natl. Acad. Sci. U. S. A.* **2002**, *99*, 15112–7, doi:10.1073/pnas.232349399.

Western blot original images

Figure 1b

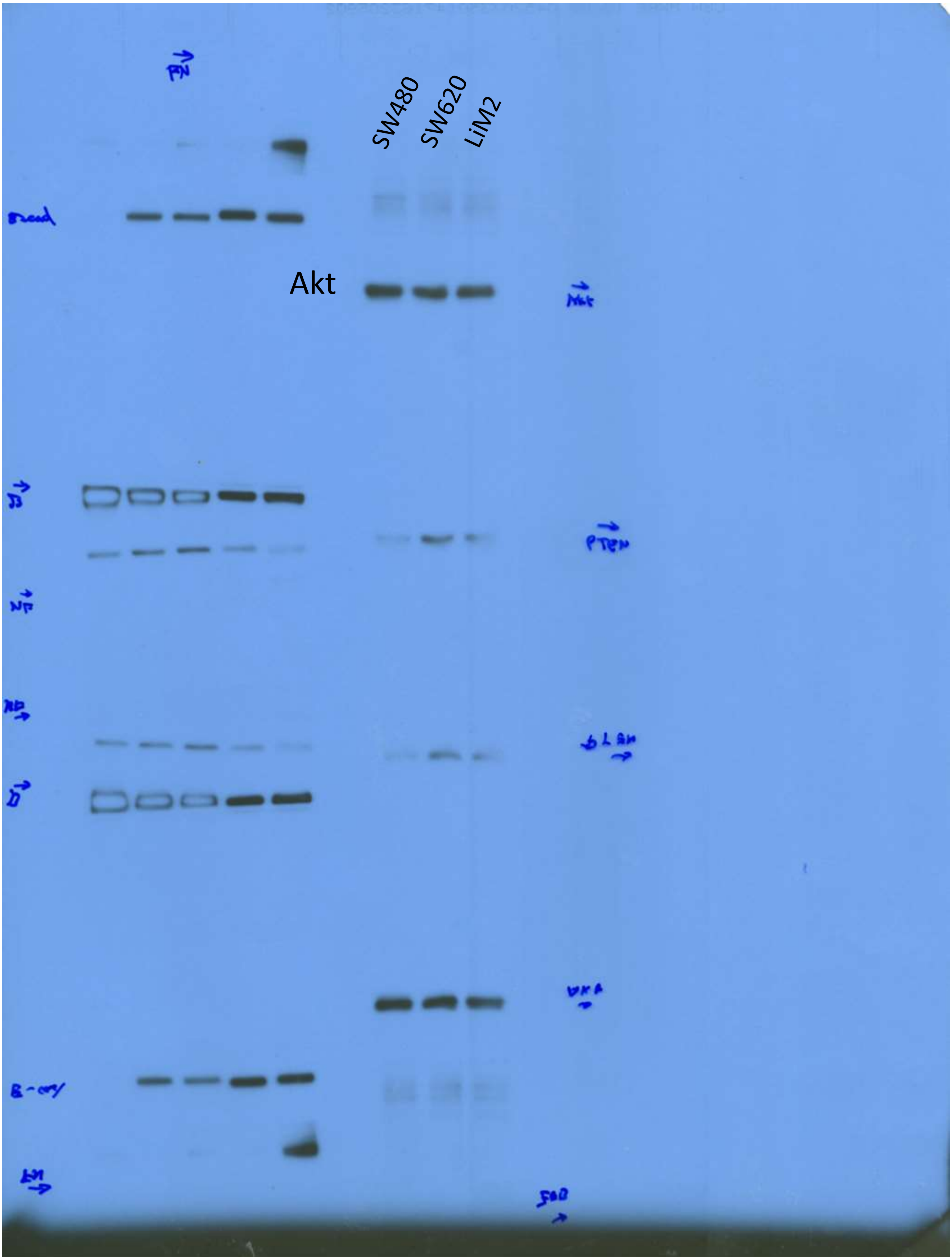

Figure 1b

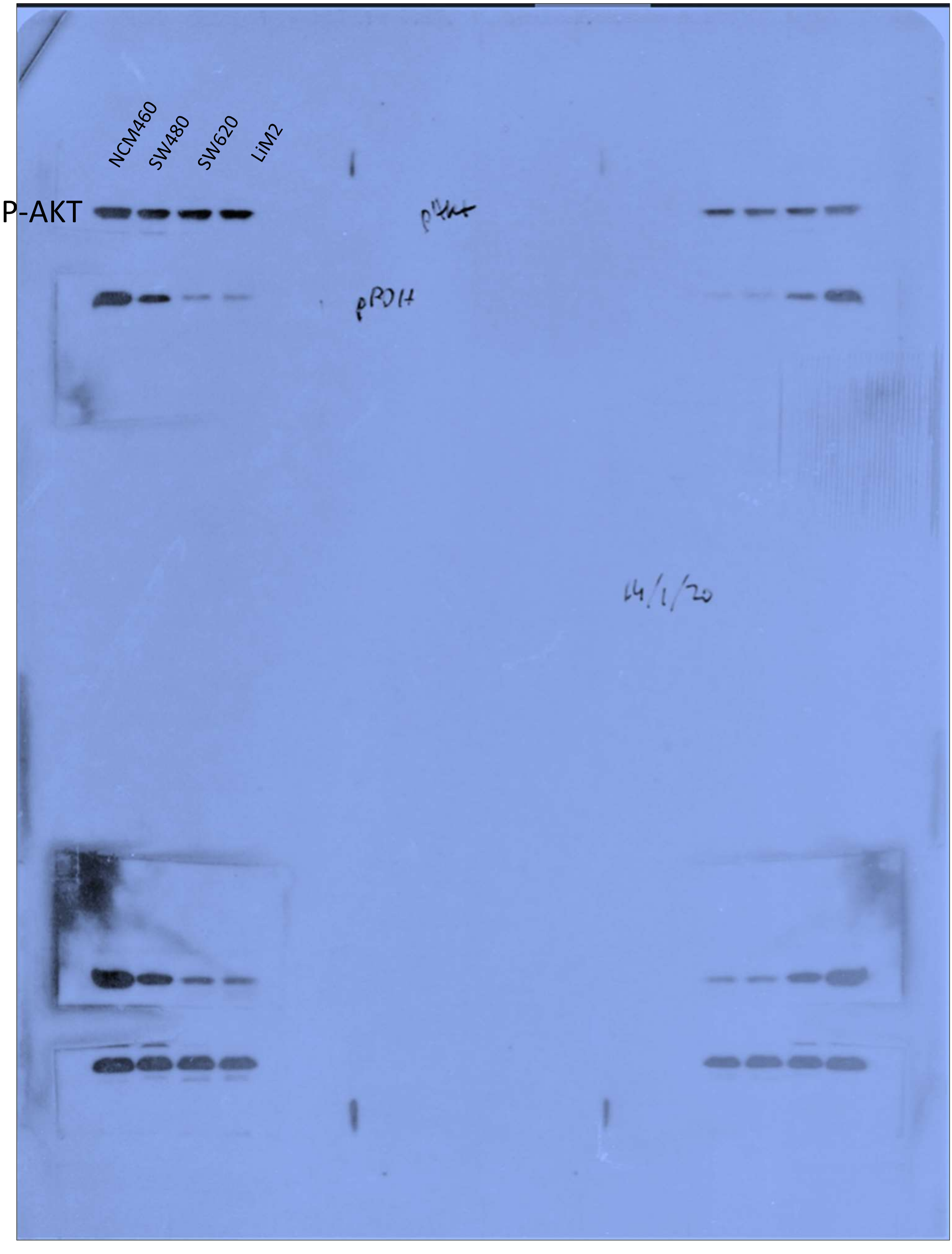

Figure 1b

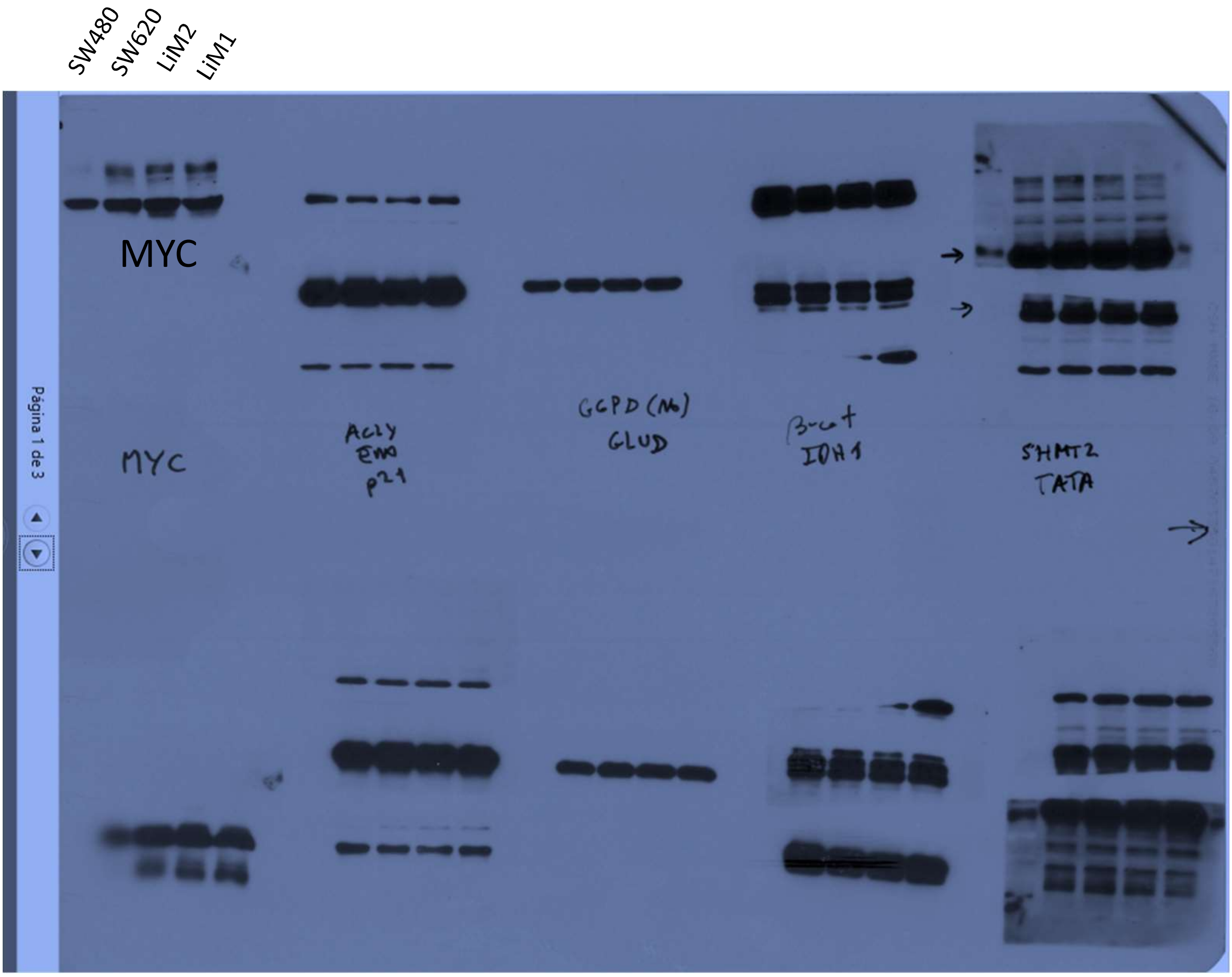
SW480
SW620
LiM2
LiM1
MYC
MYC
Acly
Eno
p21
G6PD (Nb)
GLUD
β-cat
IDH1
SHMT2
TATA
Página 1 de 3

Figure 1b

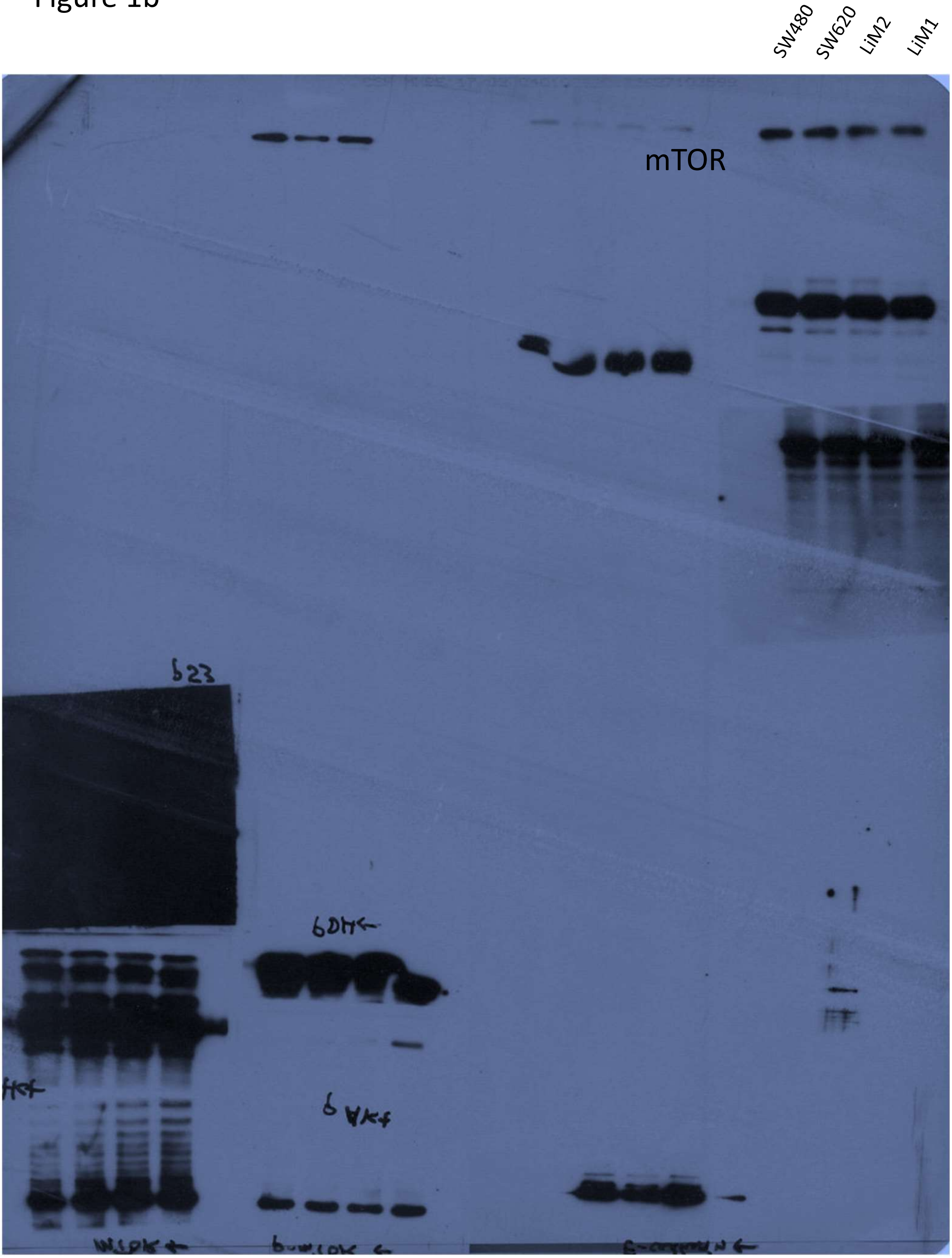

Figure 1b

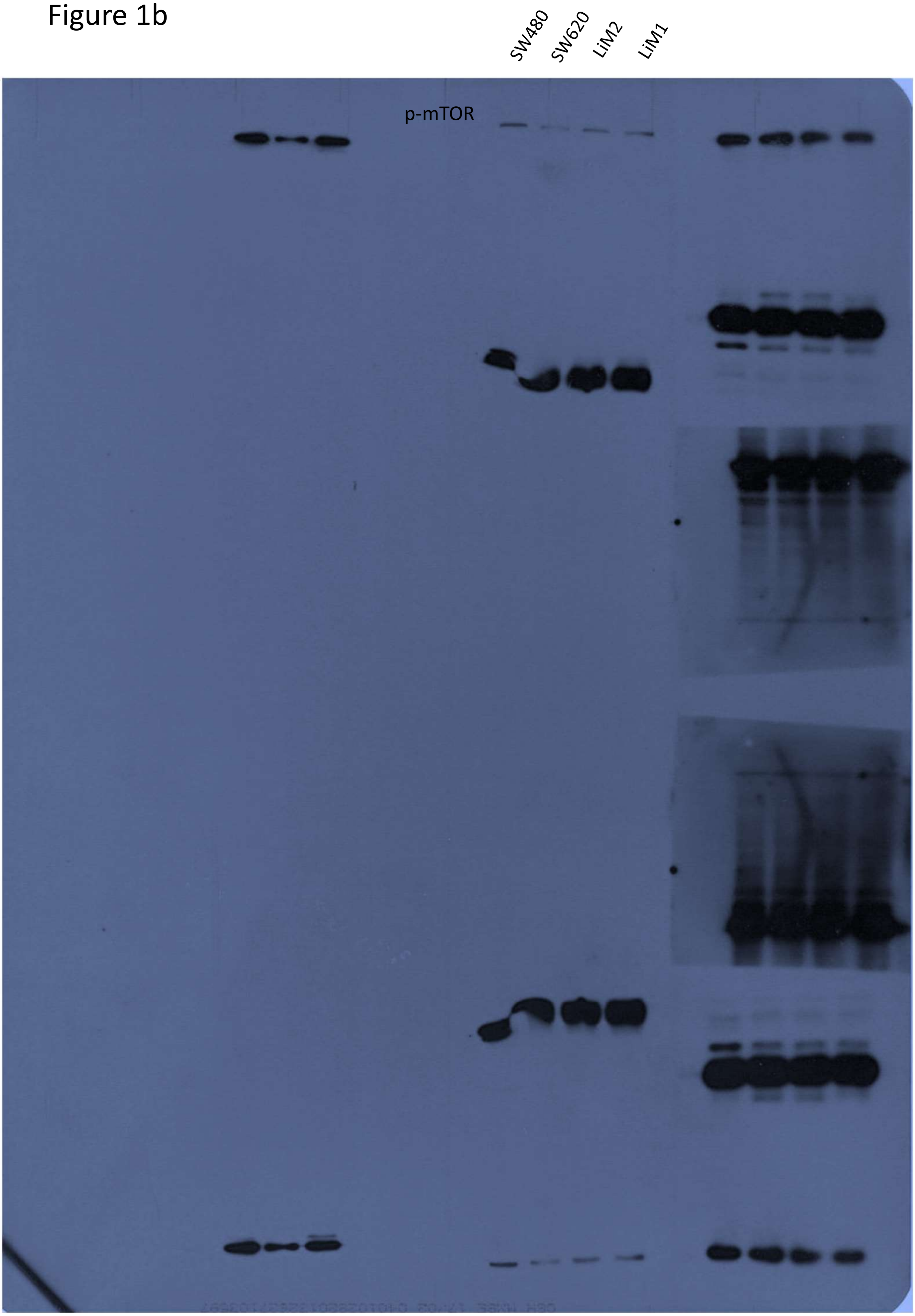

Figure 1b

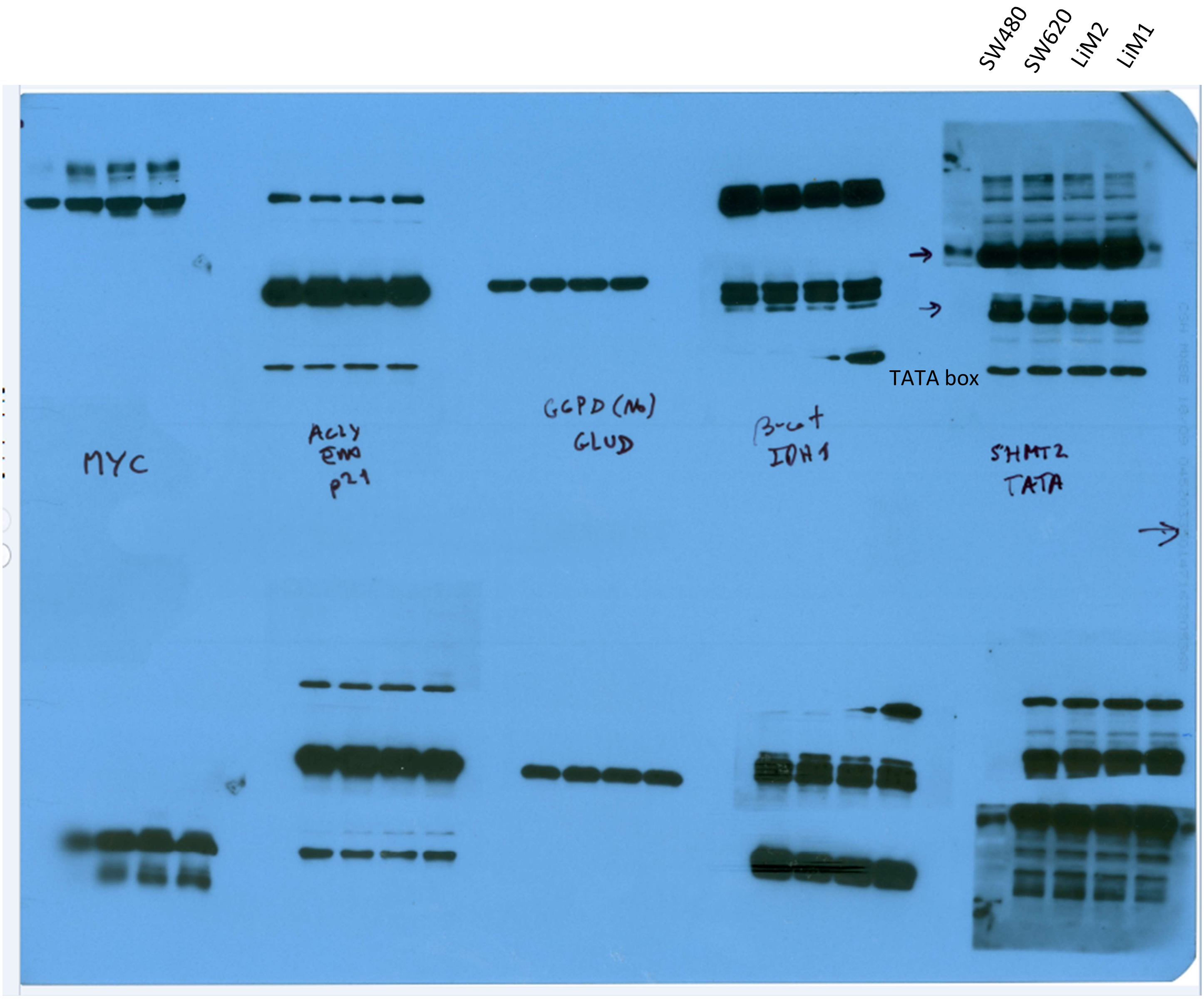
SW480
SW620
LiM2
LiM1
TATA box
MYC
Acly
G6PD (No)
GLUD
IDH1
SHMT2
TATA

Figure 1g

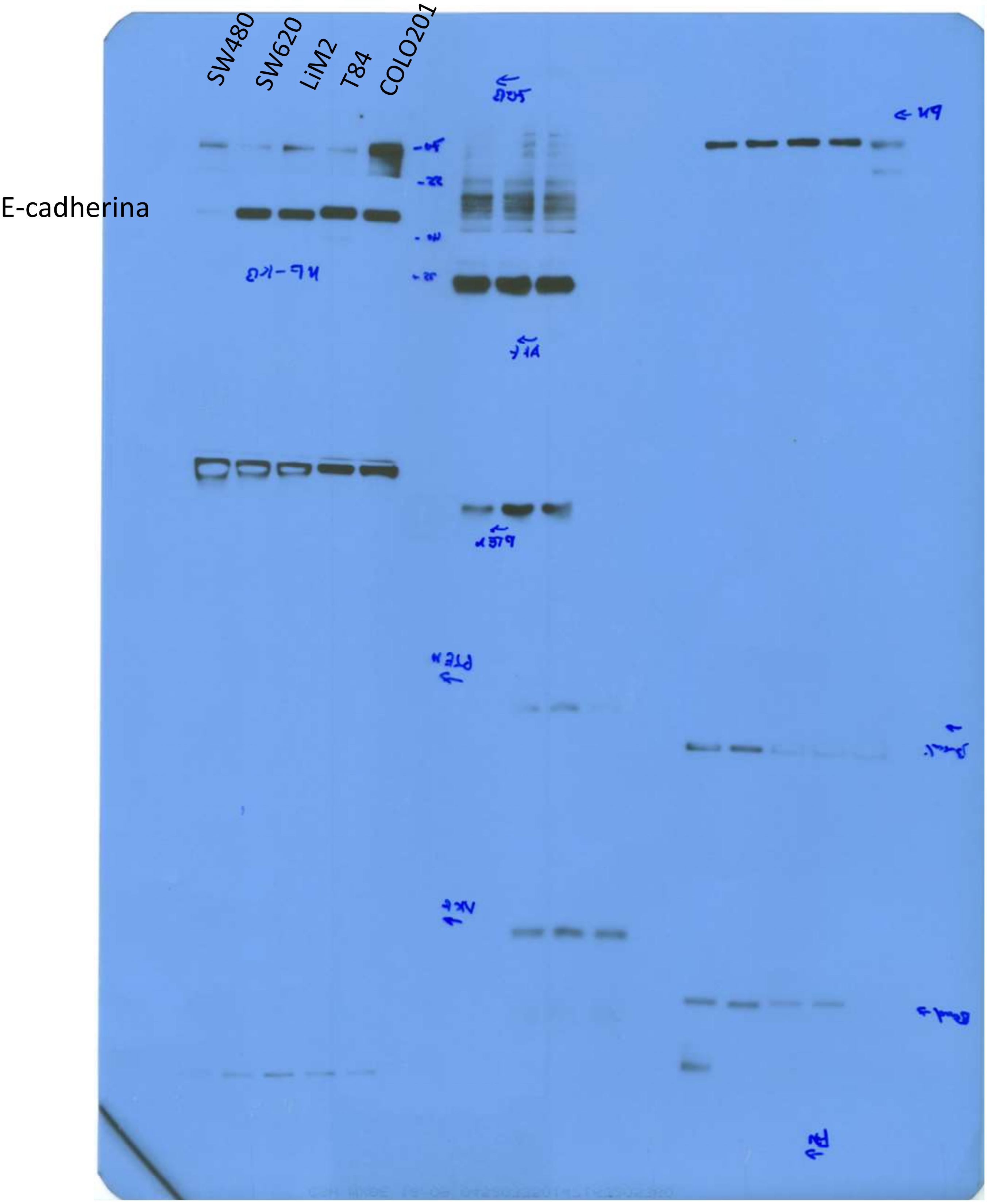

Figure 1g

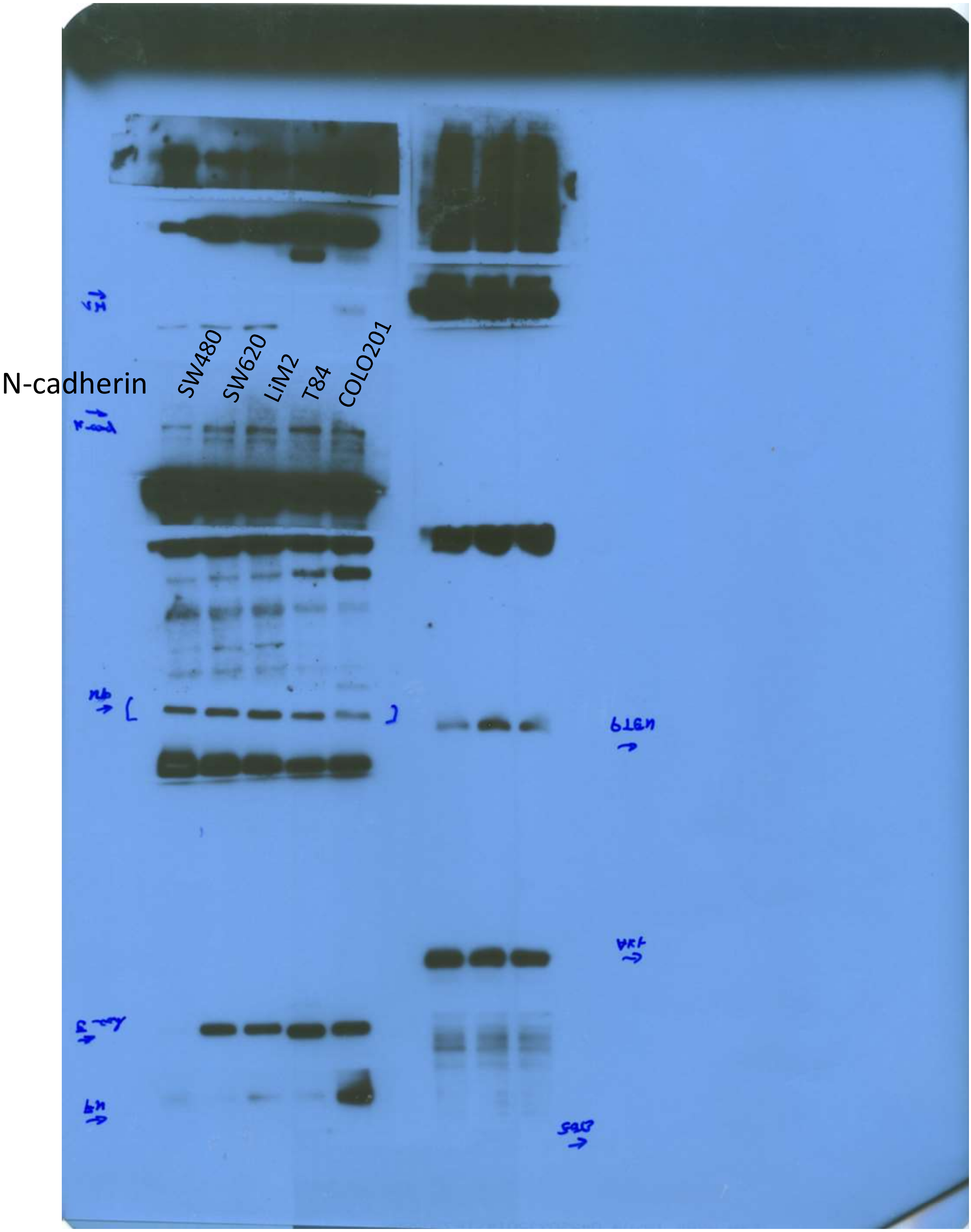

Figure 1g

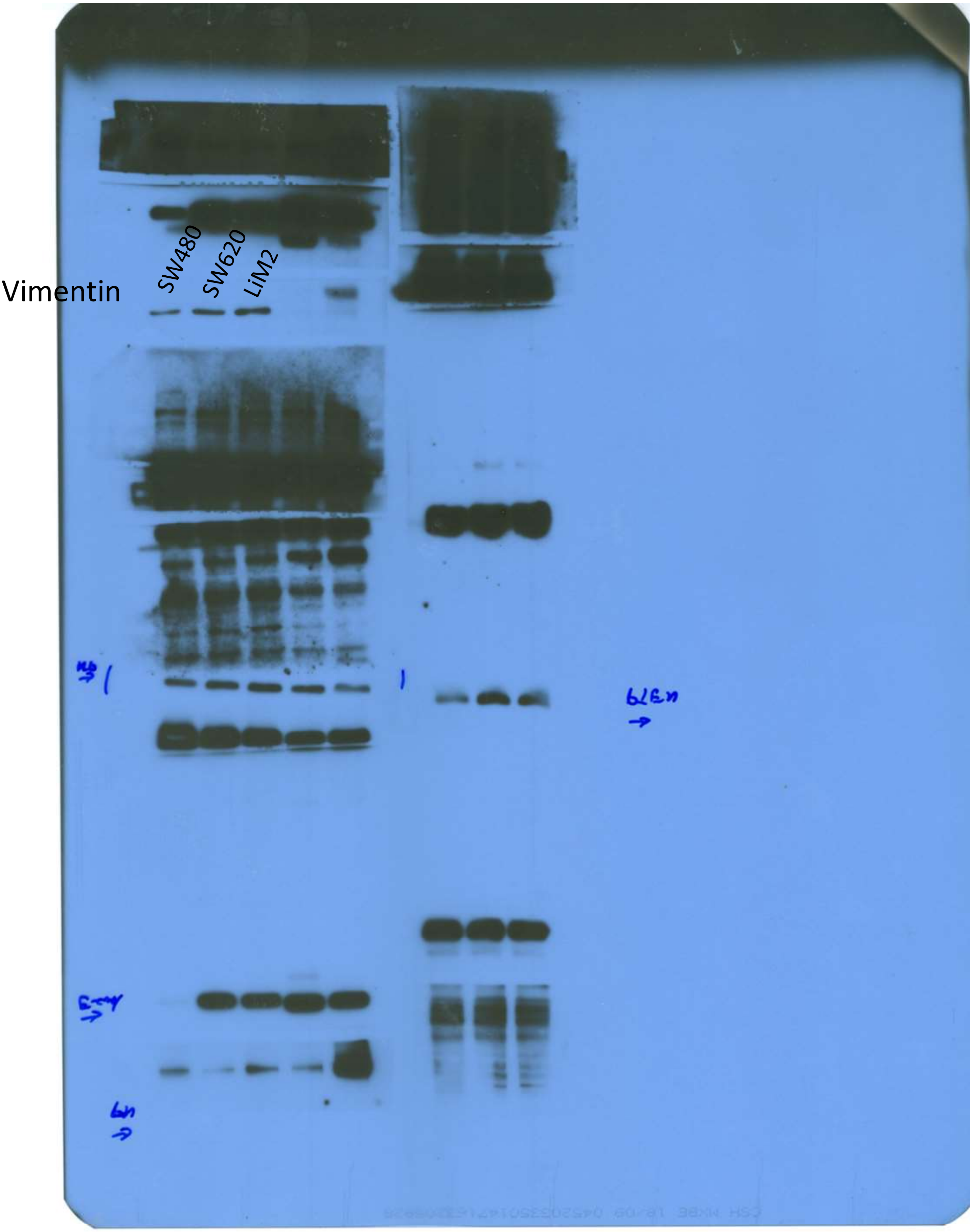

Figure 1g

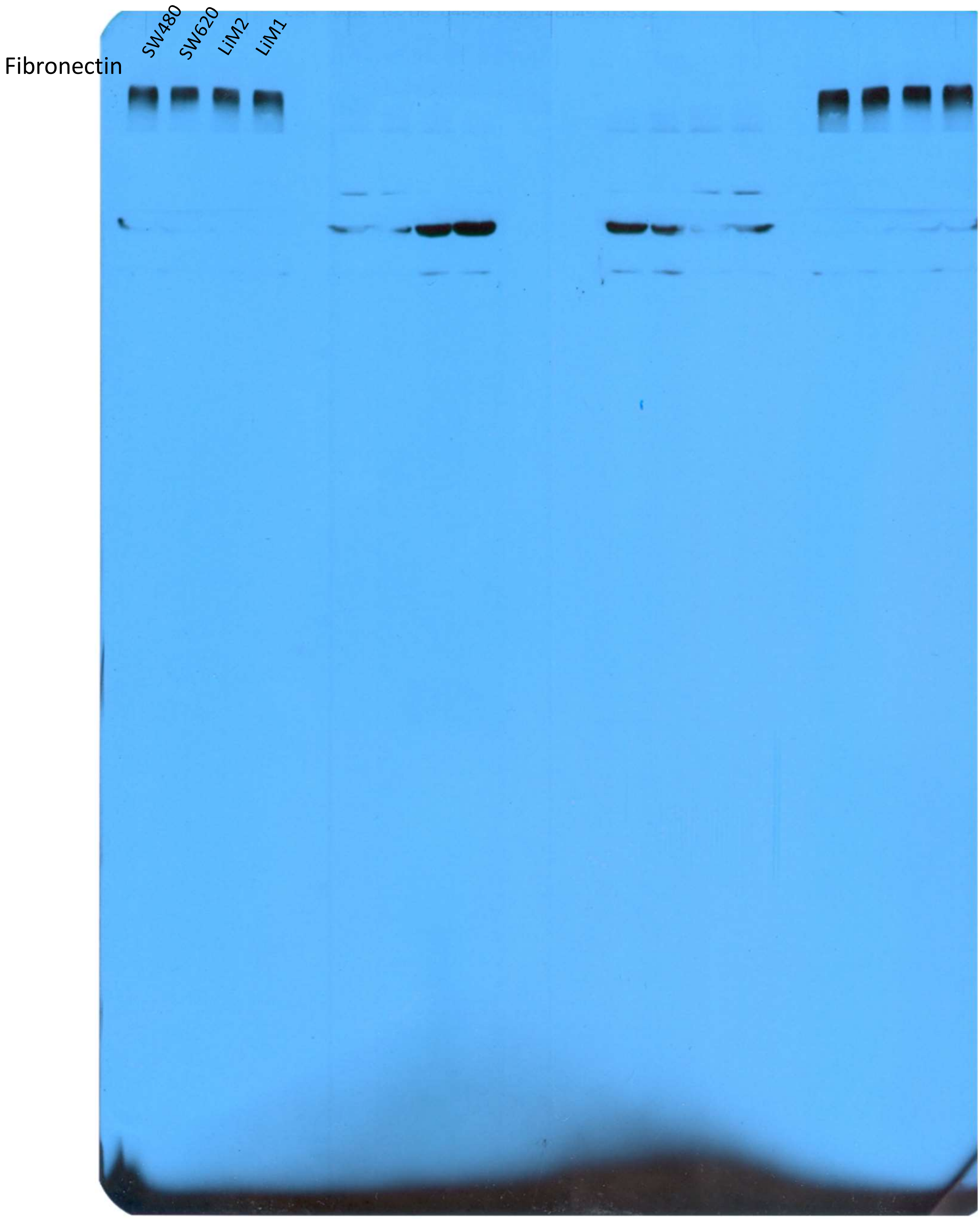

Figure 1g

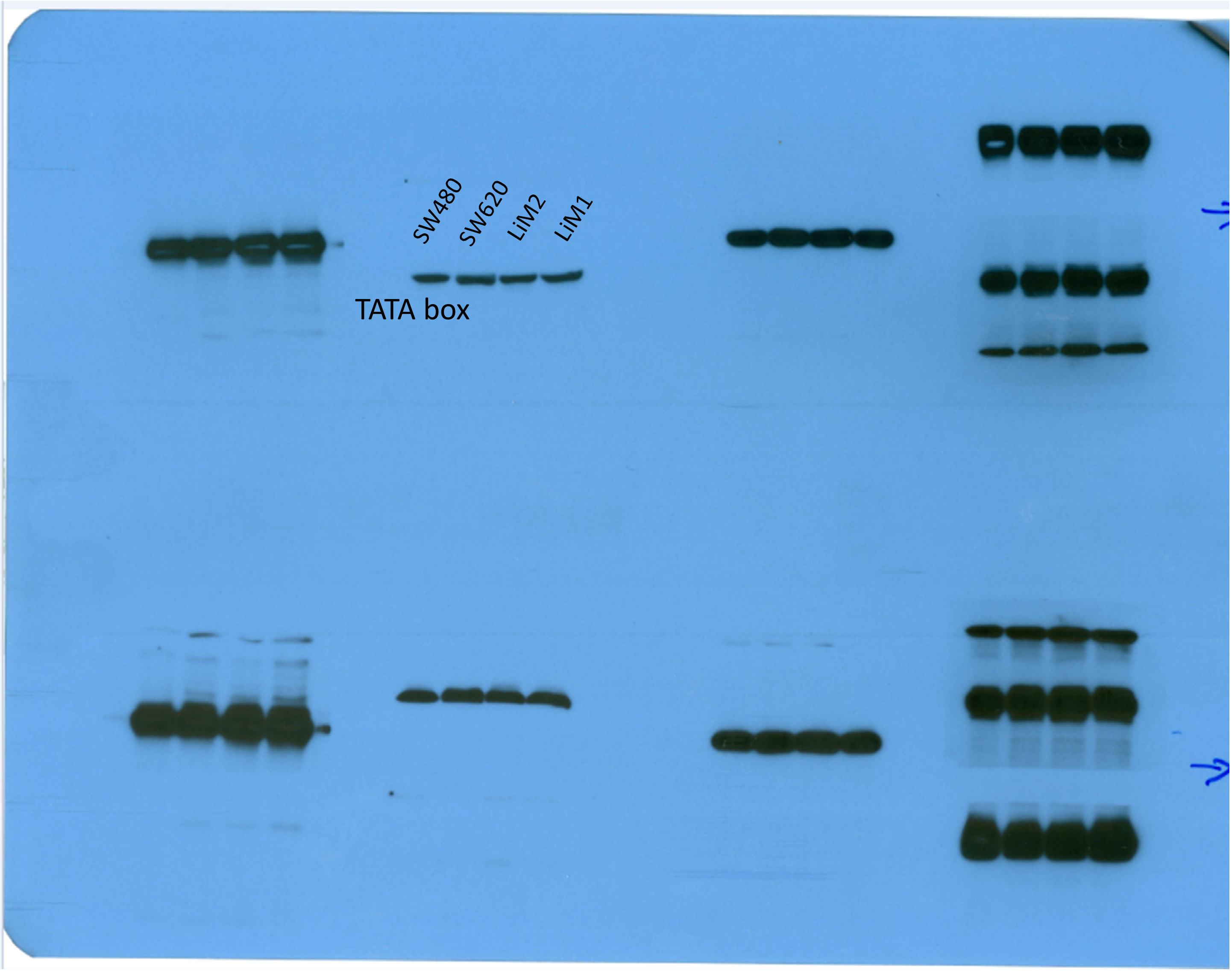

Figure 1h

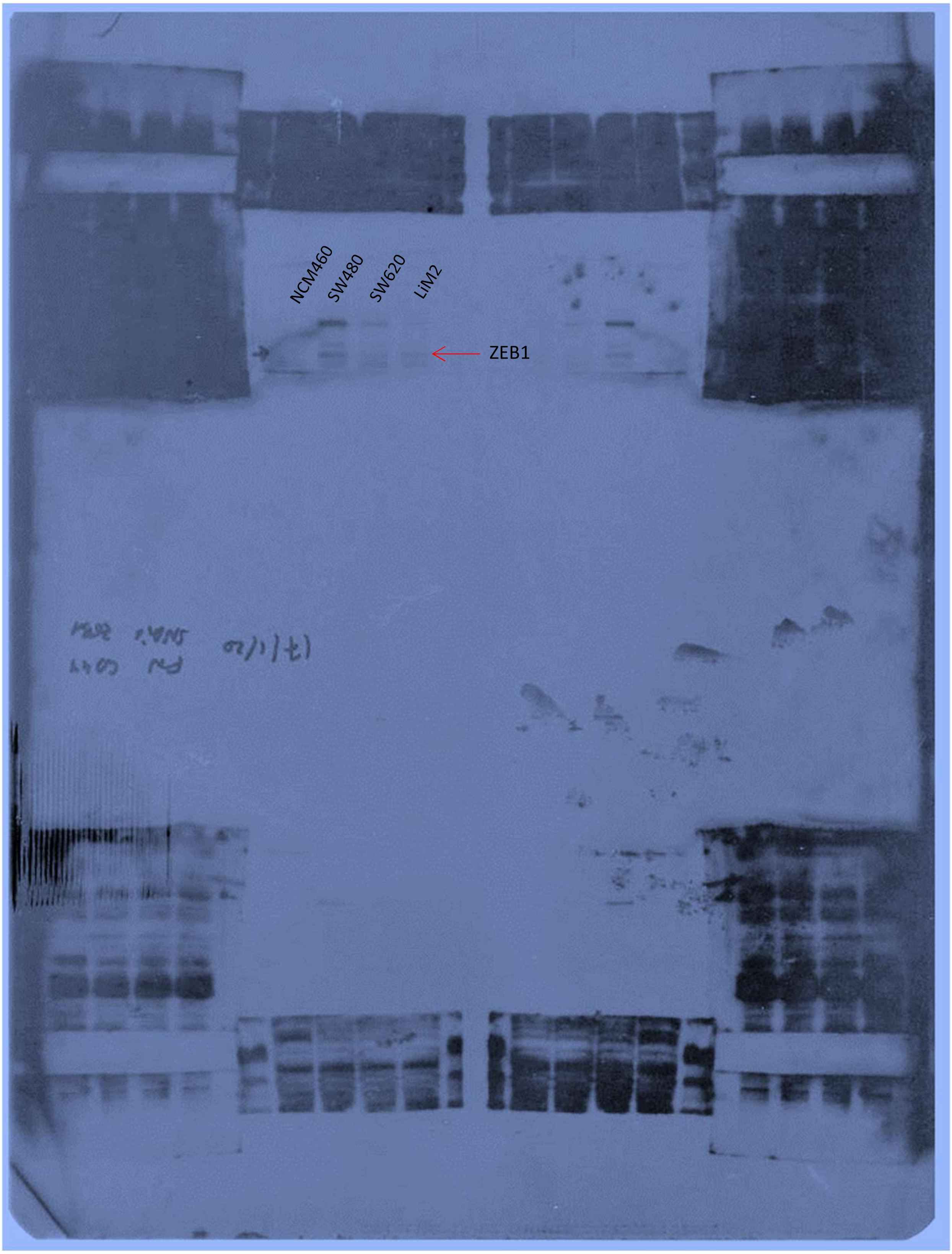

Figure 1h

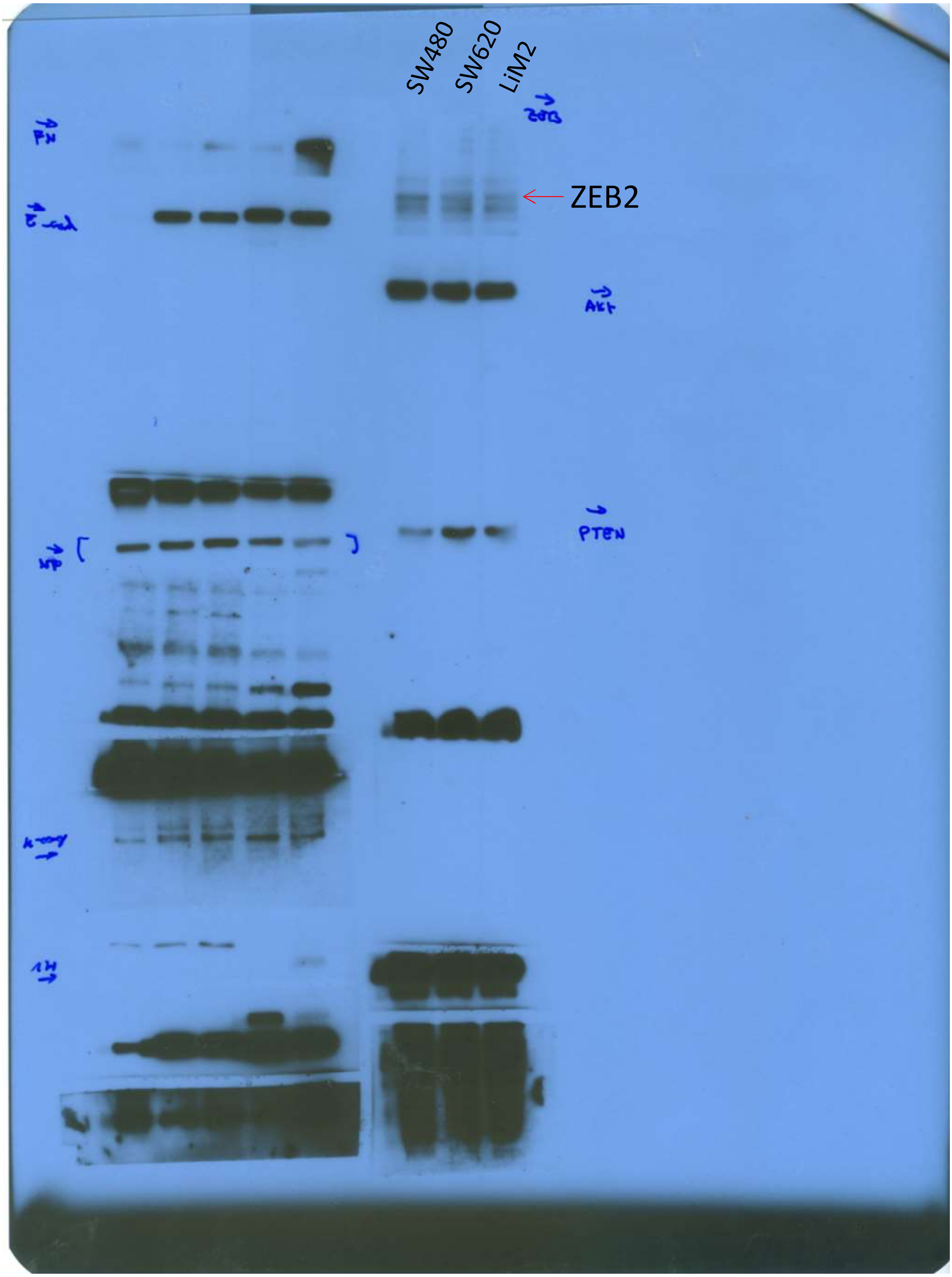

Figure 1h

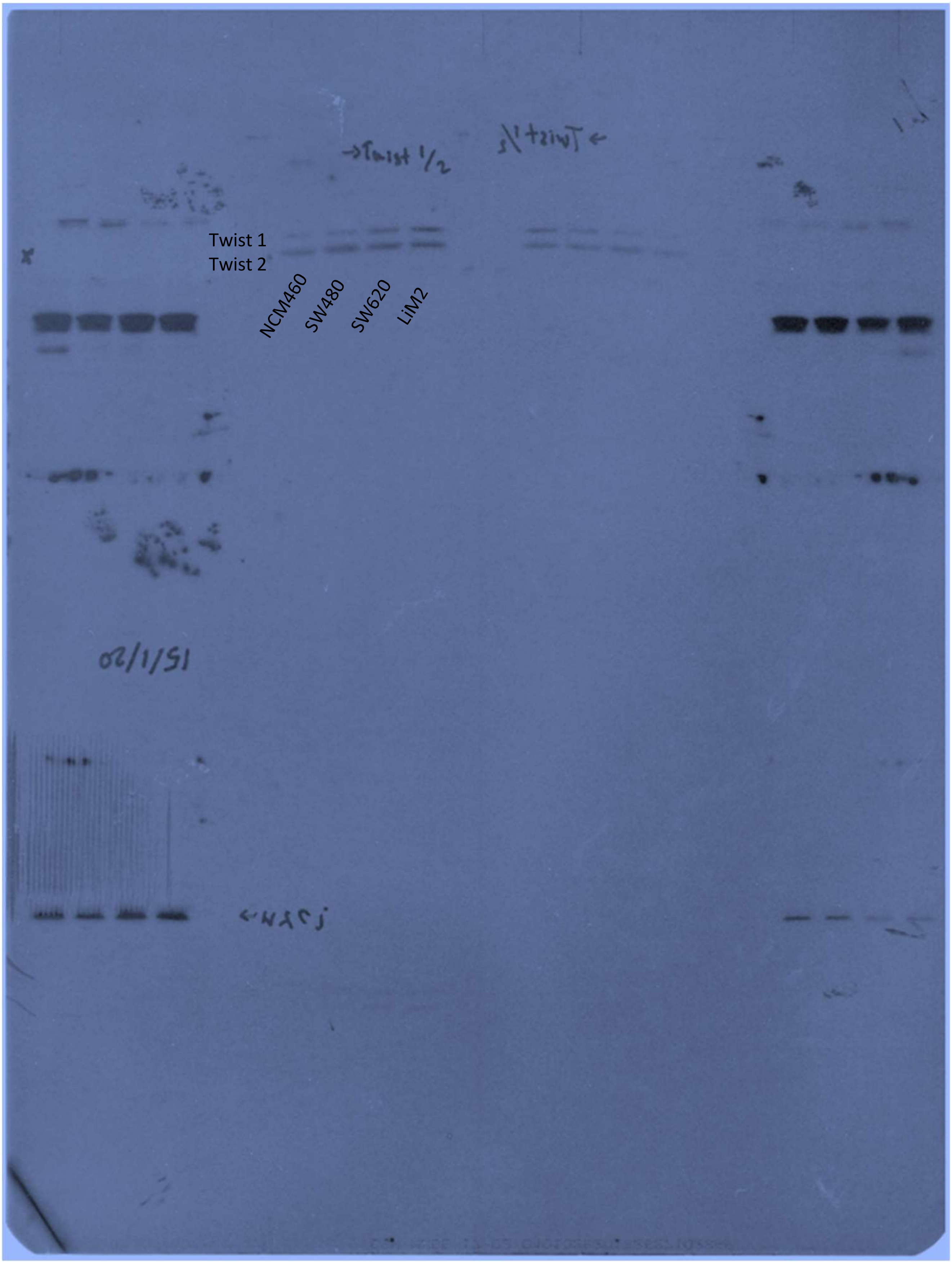

Figure 1h

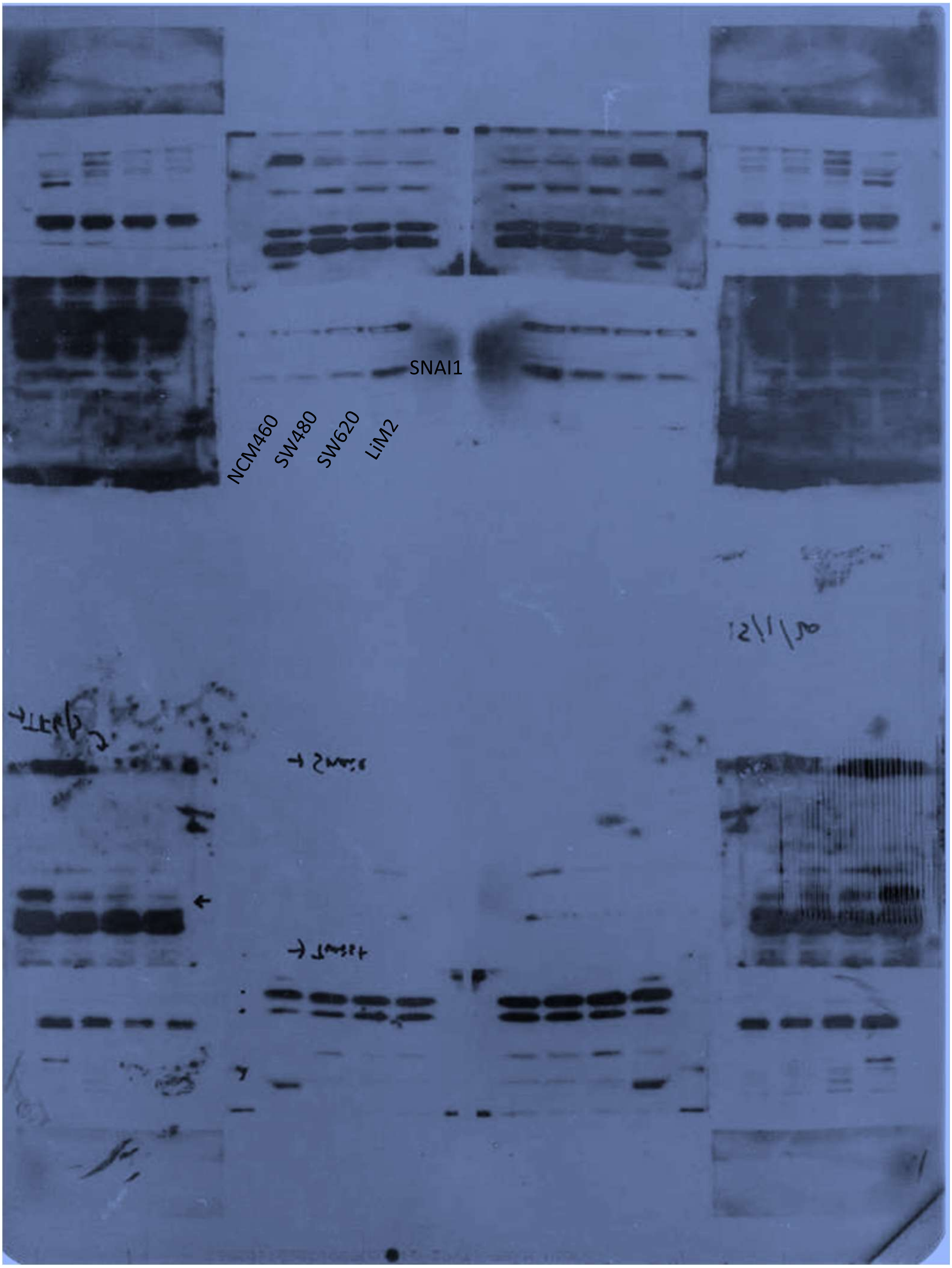

Figure 1h

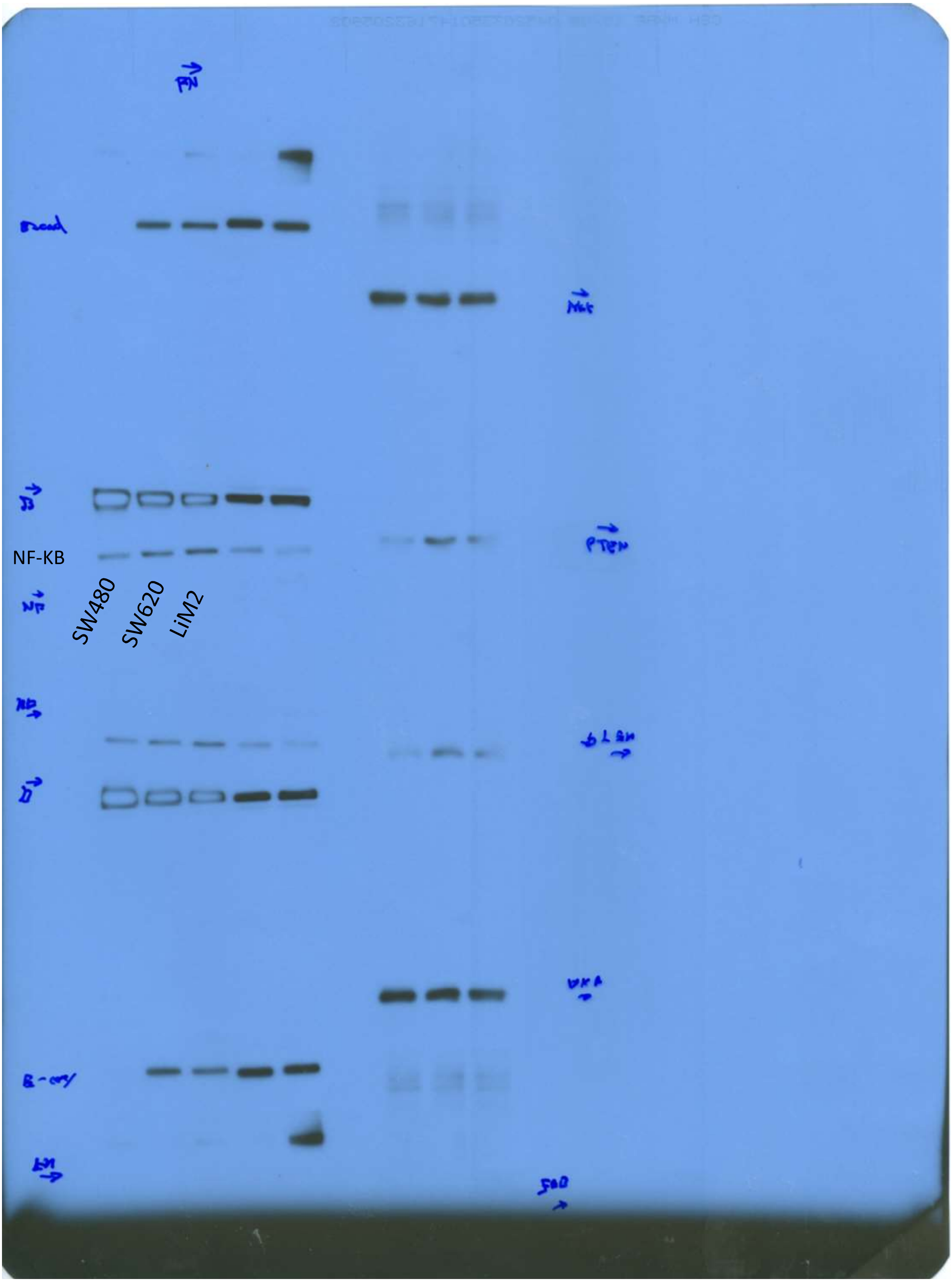

Figure 1h

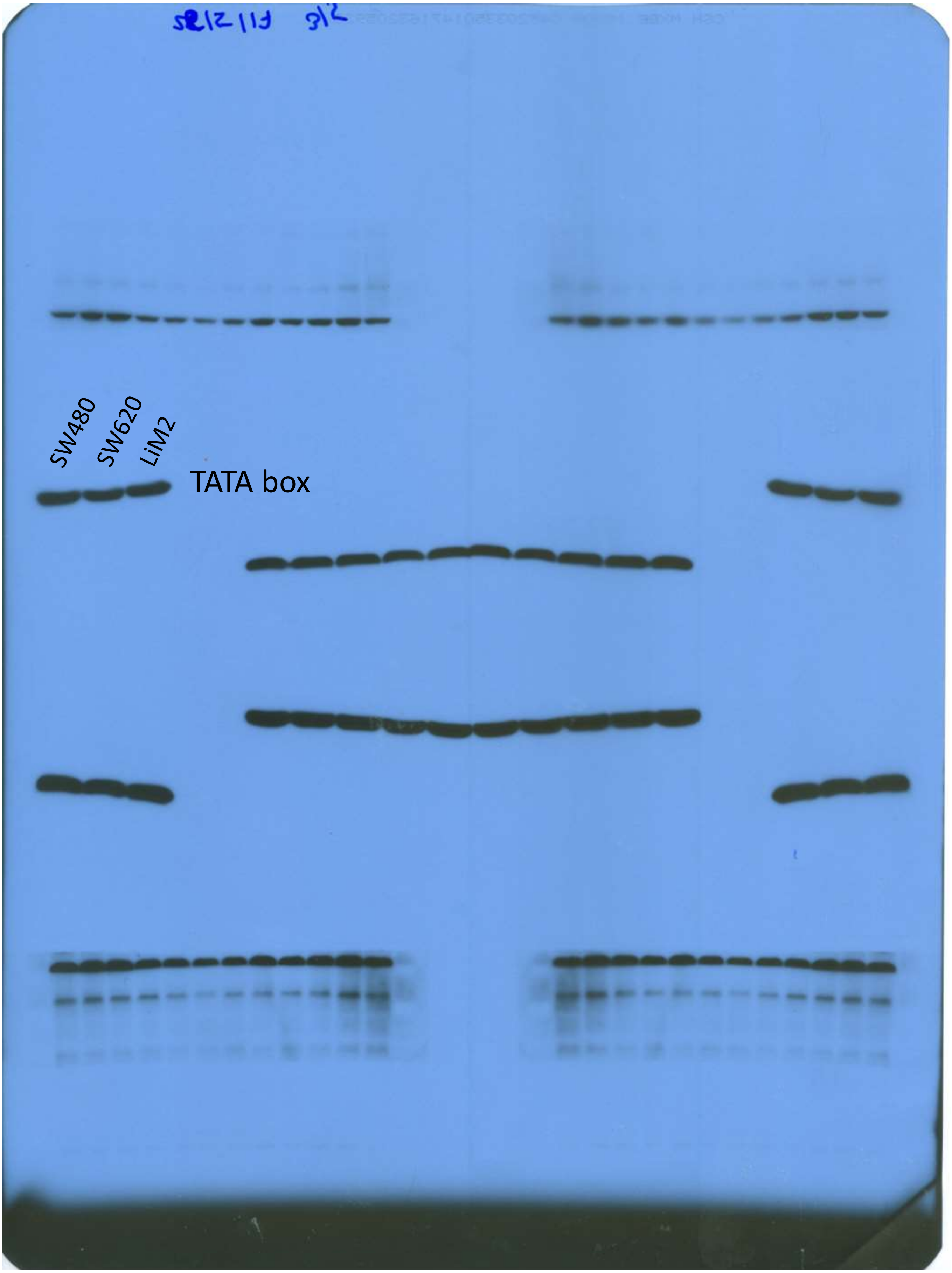

Figure 2d

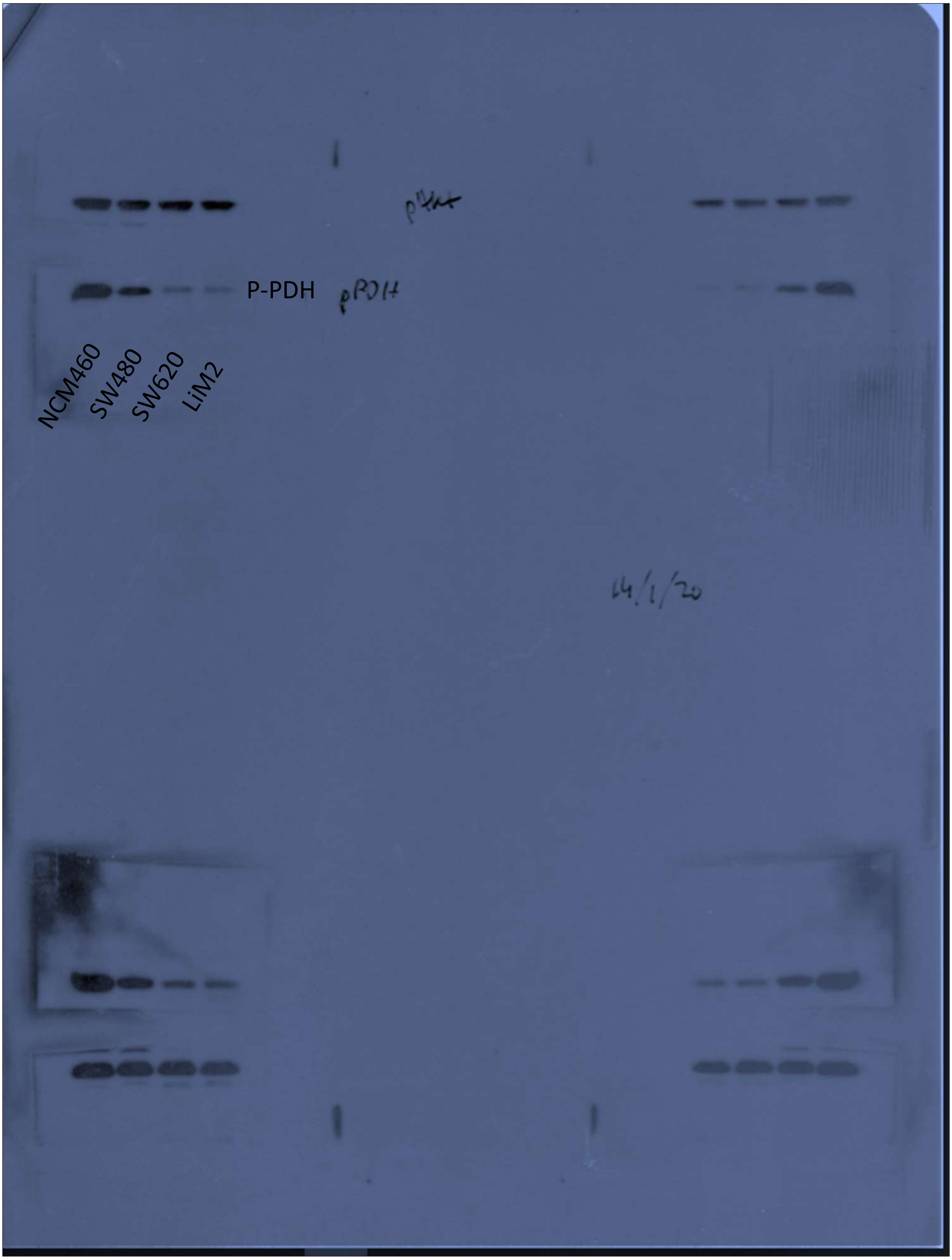

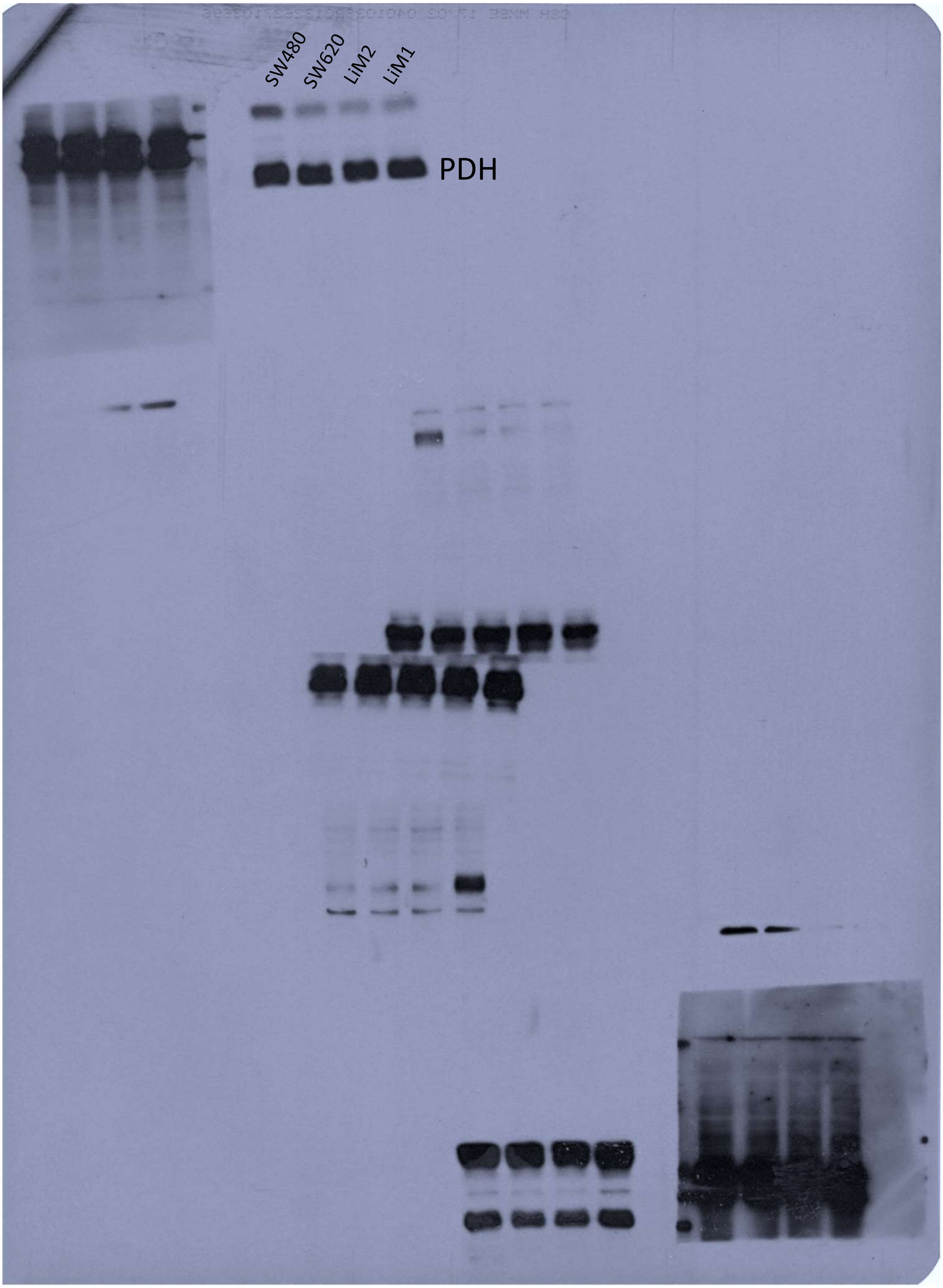

SW480
SW620
LiM2
LiM1
PDH

Figure 2d

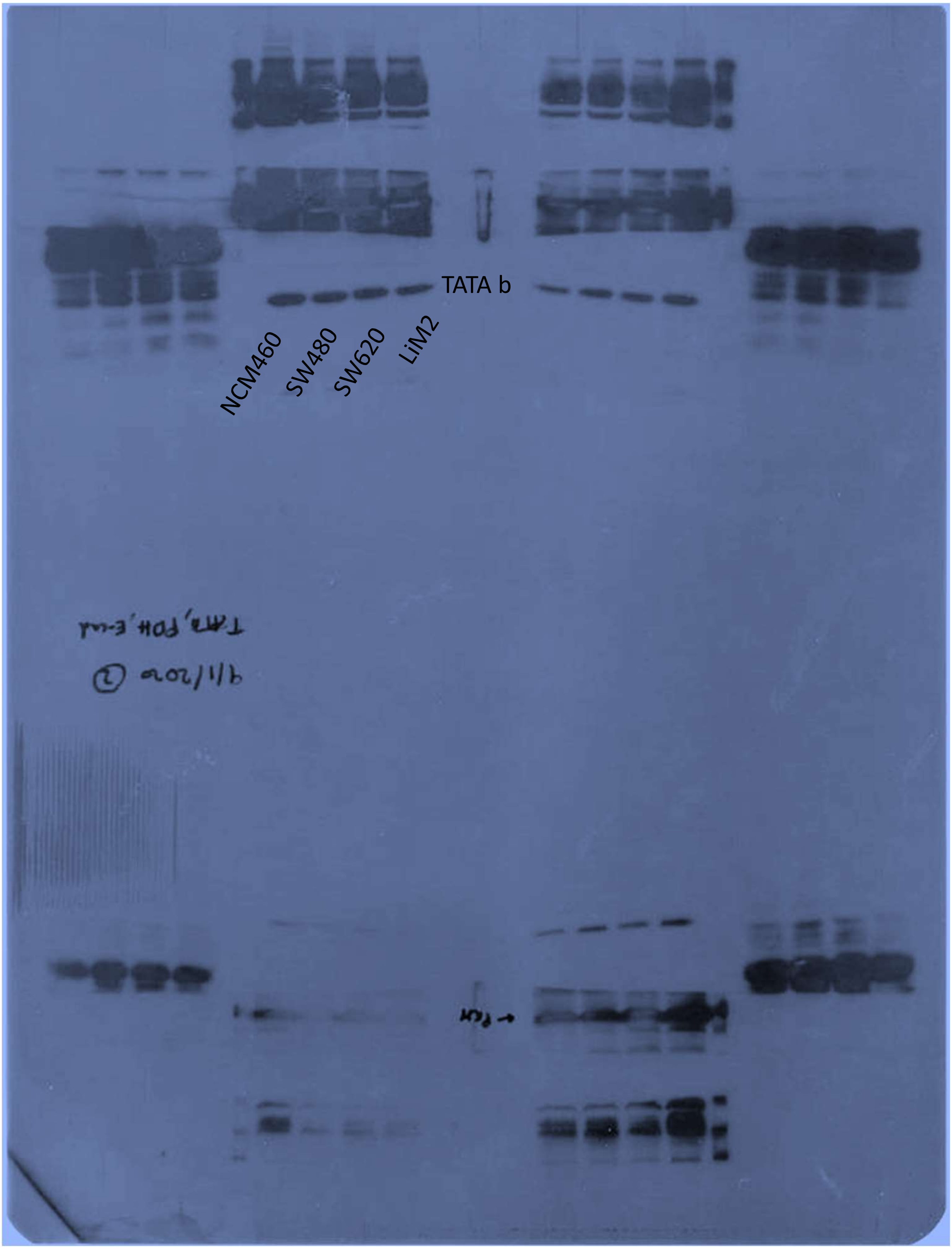

Figure 2i

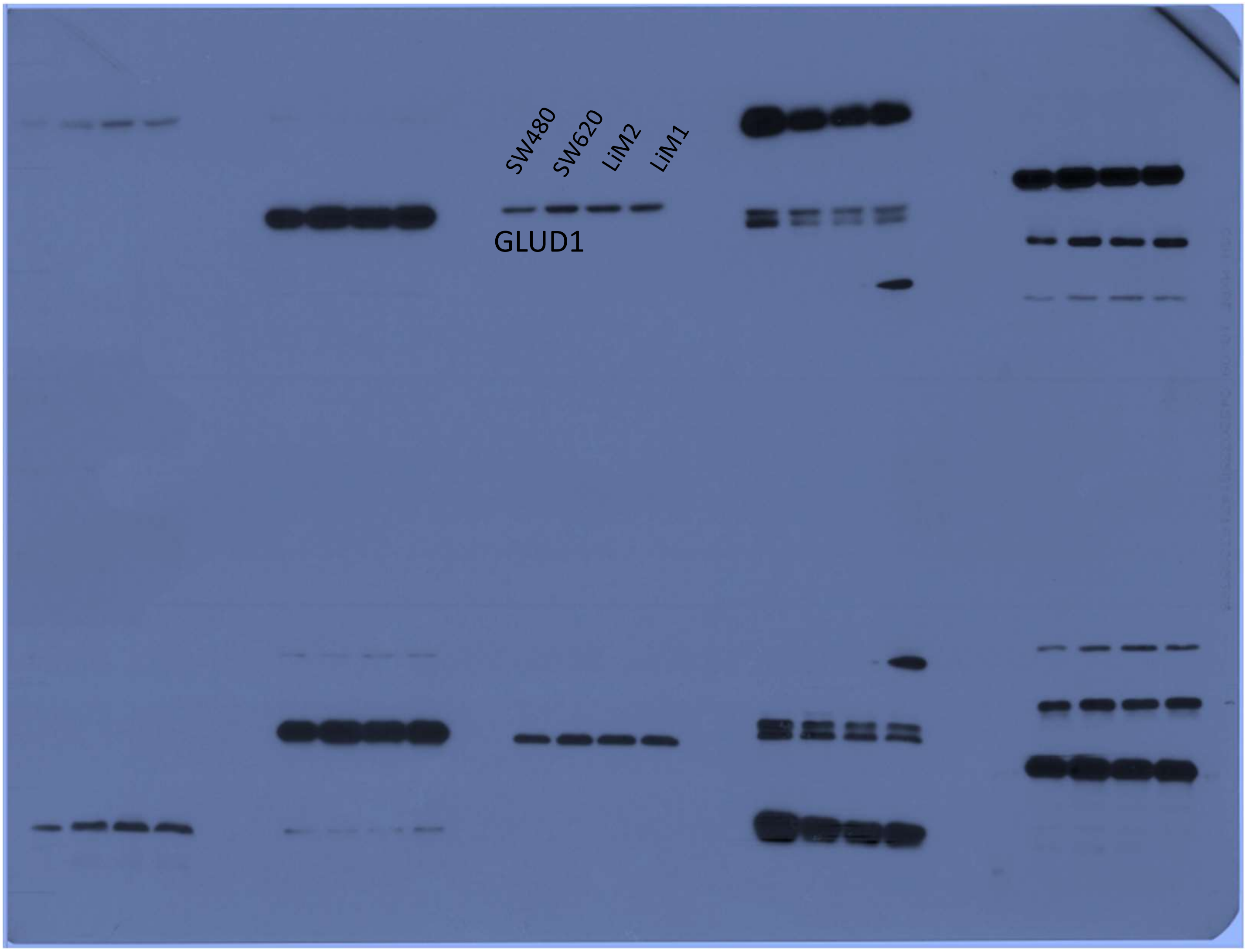

Figure 2i

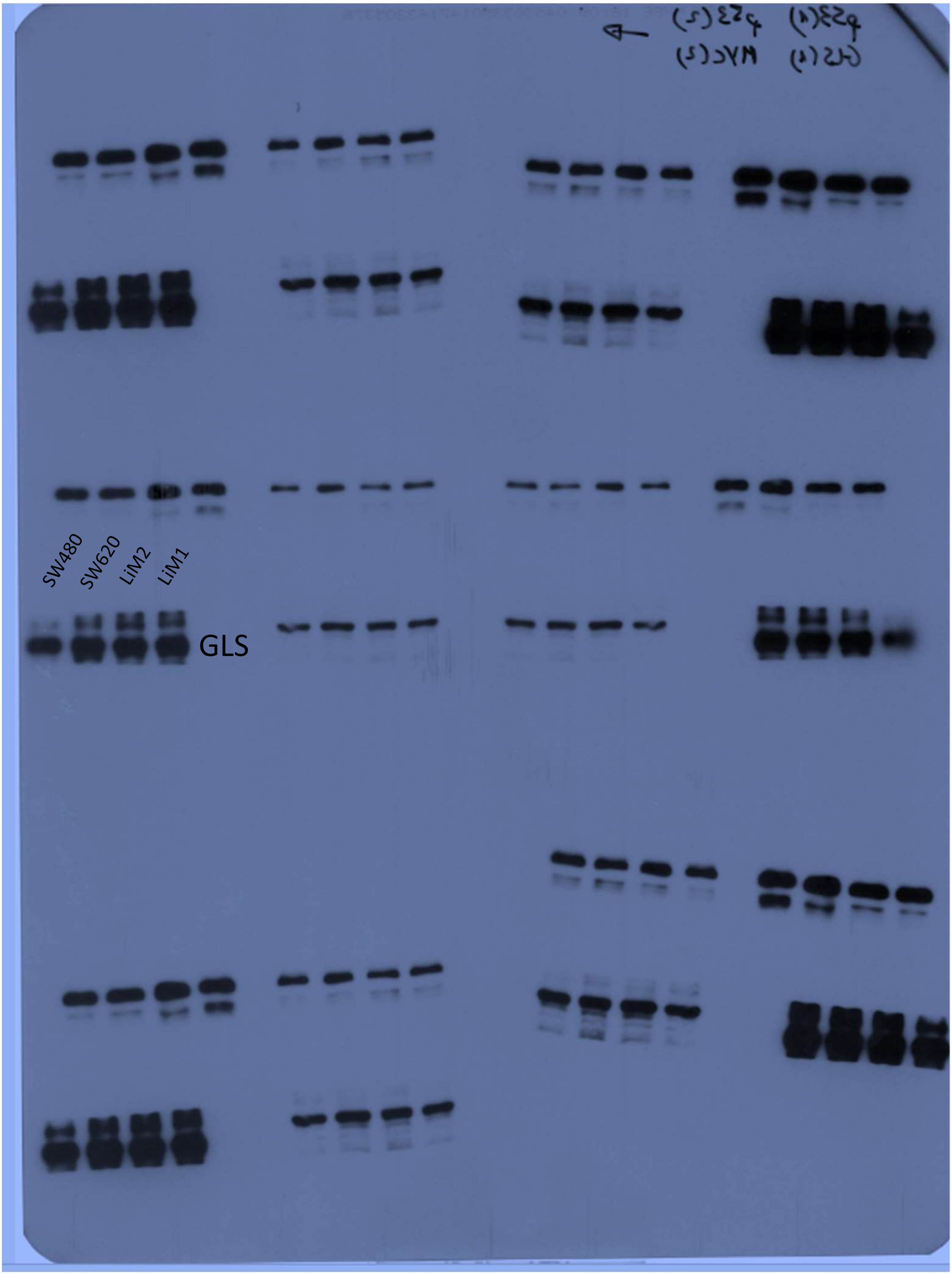

Figure 2i

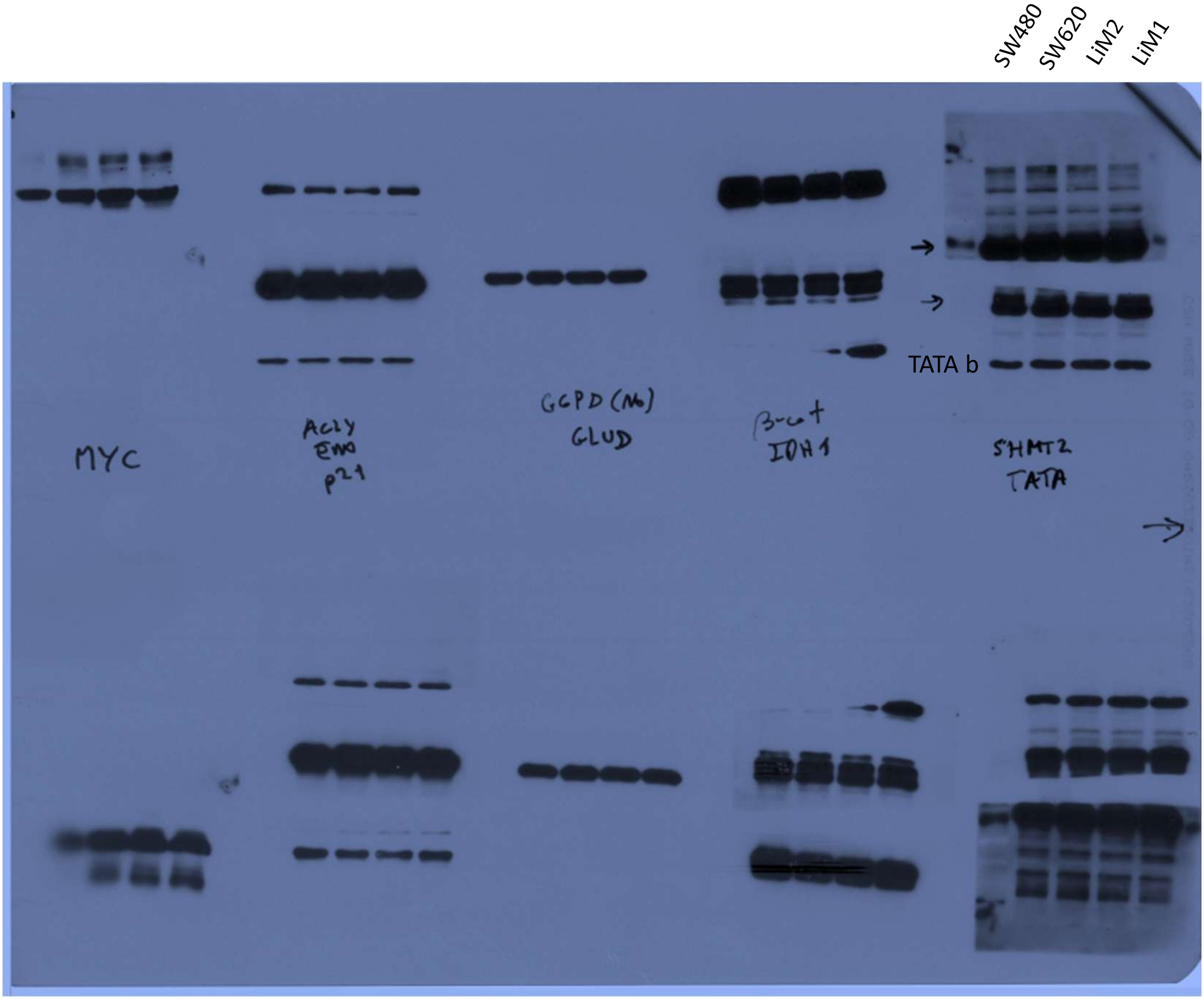
SW480
SW620
LiM2
LiM1
TATA b
MYC
Acly
Eno
p21
G6PD (Ab)
GLUD
β-cat
IDH1
SHMT2
TATA

Figure 3c

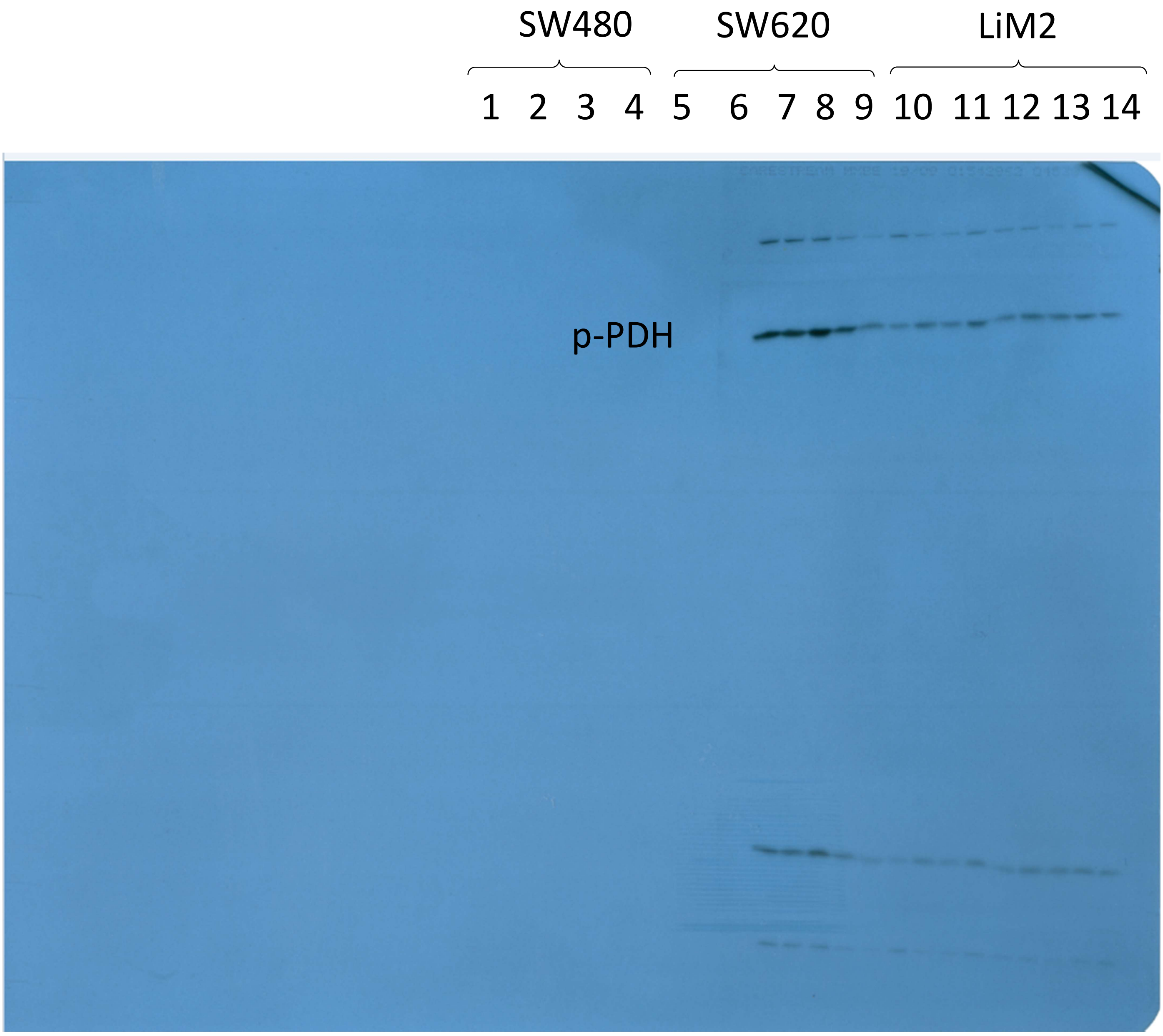

Figure 3c

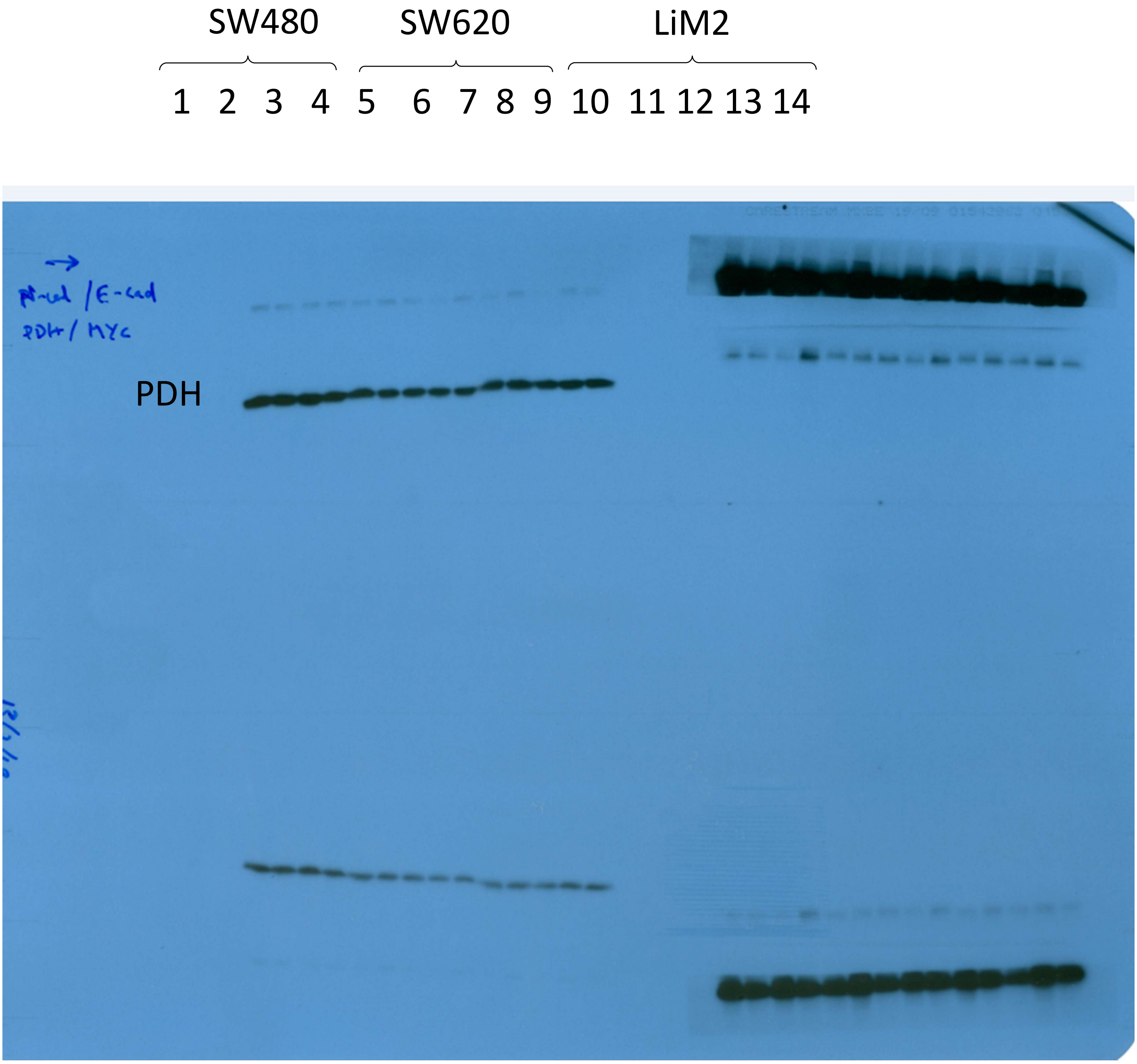

Figure 3c

SW480 SW620 LiM2

1 2 3 4 5 6 7 8 9 10 11 12 13 14

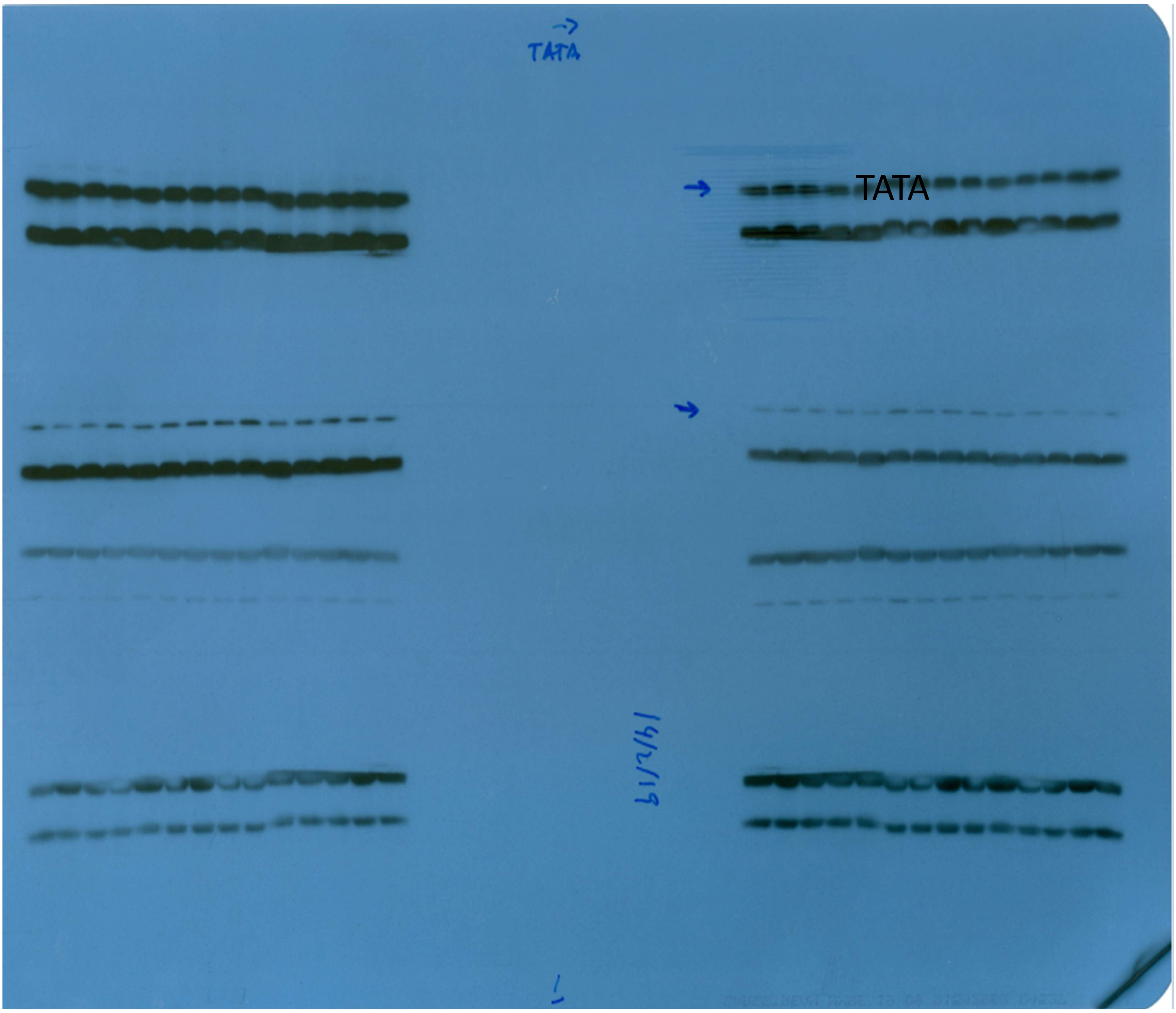

Supplement: Supplementary file 1 [file cancers-13-00425-s001.pdf]
